# D1 Dopamine Receptor Activation Induces Neuronal eEF2 Pathway-Dependent Protein Synthesis

**DOI:** 10.3389/fnmol.2020.00067

**Published:** 2020-05-15

**Authors:** Orit David, Iliana Barrera, Nathaniel Gould, Shunit Gal-Ben-Ari, Kobi Rosenblum

**Affiliations:** ^1^Sagol Department of Neurobiology, University of Haifa, Haifa, Israel; ^2^School of Physiology, Pharmacology and Neuroscience, University of Bristol, Bristol, United Kingdom; ^3^Center for Gene Manipulation in the Brain, University of Haifa, Haifa, Israel

**Keywords:** dopamine, D1 receptor, NMDA receptor, ERK, mTOR, anti-depressant, eEF2, protein synthesis

## Abstract

Dopamine, alongside other neuromodulators, defines brain and neuronal states, *inter alia* through regulation of global and local mRNA translation. Yet, the signaling pathways underlying the effects of dopamine on mRNA translation and psychiatric disorders are not clear. In order to examine the molecular pathways downstream of dopamine receptors, we used genetic, pharmacologic, biochemical, and imaging methods, and found that activation of dopamine receptor D1 but not D2 leads to rapid dephosphorylation of eEF2 at Thr^56^ but not eIF2α in cortical primary neuronal culture in a time-dependent manner. NMDA receptor, mTOR, and ERK pathways are upstream of the D1 receptor-dependent eEF2 dephosphorylation and essential for it. Furthermore, D1 receptor activation resulted in a major reduction in dendritic eEF2 phosphorylation levels. D1-dependent eEF2 dephosphorylation results in an increase of BDNF and synapsin2b expression which was followed by a small yet significant increase in general protein synthesis. These results reveal the role of dopamine D1 receptor in the regulation of eEF2 pathway translation in neurons and present eEF2 as a promising therapeutic target for addiction and depression as well as other psychiatric disorders.

## Introduction

The dopaminergic system (DS) plays an important role in reward predictions (Schultz, 1998), motivational arousal, and responsiveness to conditioned incentive stimuli (Salamone et al., 2003). In accordance, its dysfunction is associated with disruption of motivation to seek out pleasure experiences, as described in individuals diagnosed with mood disorders such as depression (Sherdell et al., 2012). Dopamine acts via two different metabotropic receptors, D1 and D2, that induce different signal transduction and cellular phenotypes (Boyd and Mailman, 2012). Indeed, recently, Hare et al. (2019) demonstrated that D1 receptors (D1R) in the medial prefrontal cortex (mPFC) might contribute to the antidepressant-like effects of ketamine (Hare et al., 2017; Hare et al., 2019). While it is established that one mechanism through which dopamine affects brain and neuronal states is through the regulation of mRNA translation, the signaling pathways mediating this regulation are not known. Since we and others have previously shown that the fast antidepressant effect of ketamine is mediated by the reduction in eEF2 phosphorylation (Duman and Voleti, 2012; Adaikkan et al., 2018), here, we tested the hypothesis that dopamine regulates mRNA translation regulation through the eukaryotic elongation factor 2 (eEF2).

The eEF2 translation factor and its sole known kinase (eEF2K) (Kenney et al., 2015) play a central role in the regulation of the elongation phase of mRNA translation (Montanaro et al., 1976). This regulation is mediated by eEF2K, which phosphorylates eEF2 on Thr^56^ and thereby inactivates it, leading to reduction in the rate of mRNA translation. Since eEF2K activity is regulated by Ca^2+^/calmodulin, elevation in intracellular calcium by synaptic activation receptors such as NMDA or G-coupled receptors (e.g. metabotropic glutamate receptors) results in induced neuronal activity-dependent phosphorylation of eEF2 (Sutton et al., 2007; Barrera et al., 2008; Im et al., 2009; Proud, 2015; Heise et al., 2017). Furthermore, general translation is reduced in dendrites due to eEF2K activity, while certain synaptic proteins are selectively translated at the synapse (Heise et al., 2014). eEF2K can be deactivated by other kinases such as p70S6 kinase (S6K) or p90 ribosomal kinase (p90 RSK), which are activated in response to changes in mammalian target of rapamycin-signaling (mTOR) or extracellular-regulated kinase (ERK) signaling, respectively. These kinases inactivate eEF2K by phosphorylation on its Ser366 residue (Redpath et al., 1993; Wang et al., 2001; Browne et al., 2004).

In this work, we show for the first time that indeed dopamine regulates mRNA translation through its effect on the eEF2 pathway in neurons. Specifically, we found that D1 but not D2 receptor activation increases protein synthesis by eEF2 dephosphorylation. This increase in protein synthesis in cortical neurons is mediated by the D1 receptor and requires NMDA receptor-dependent activation of the MEK/mTOR signaling pathways that lead to inactivation of eEF2K by phosphorylation on Ser^366^ residue resulting in eEF2 dephosphorylation. Furthermore, using eEF2K knockout mice, we show that eEF2K/eEF2 is the main pathway for D1-dependent increase in protein synthesis.

## Materials and Methods

### Animals

eEF2K-KO mice, in which coding exons 7, 8, 9, and 10 of eEF2K were deleted, were generated by the laboratory of Christopher G. Proud. We derived eEF2K wild-type (WT) and KO littermates by crossing heterozygous mice as previously described (8), eEF2K mice were bred from colonies maintained at the University of Haifa. C57BL/6 mice were obtained from local vendors (Envigo RMS, Jerusalem, Israel) and after acclimation to the facility were used for experiments. Animals were provided *ad libitum* with standard food and water and were maintained on a 12/12 h light/dark cycle. All experiments were approved by the Institutional Animal Care and Use Committee of the University of Haifa, and adequate measures were taken in order to minimize pain, in accordance with the guidelines laid down by the European Union and United States NIH regarding the care and use of animals in experiments.

### Cortical Cell Culture

Primary cortical neuronal cultures were isolated from P0 or P1 C57BL/6J or eEF2K-WT or KO mice of either sex as previously described (Ounallah-Saad et al., 2014). Briefly, both hippocampi were removed, and cortical regions were taken. The tissue was chemically dissociated by trypsin and DNase, and mechanically, using a siliconized Pasteur pipette. Cells were plated onto round coverslips coated with 20 μg/ml Poly-Lysine and 3 μg/ml laminin (Sigma), placed in 6-well plates (300,000 cells per well) or 12-well plates (150,000 cells per well). Culture medium consisted of MEM (Gibco), 25 μg/ml insulin (Sigma), 27.8 mM glucose (Sigma), 2 mM L-glutamine (Sigma), and 10% horse serum (Biological Industries, Israel). Cultures were maintained at 37°C in a 95% air/5% CO2 humidified incubator. Half the volume of the culture medium was replaced at days 8 and 11 with feeding medium containing glutamine 2 mM, insulin 25 μg/ml, and 2% B-27 supplement (Gibco).

### Pharmacological Manipulations on Primary Cortical Neurons

After 14 days *in vitro* cortical neurons were treated with dopamine D1 receptor agonist SKF38393 (25 μM, Sigma) or the D2 agonist quinpirole (10 μM, Sigma) in a time-dependent manner. Cells were pre-incubated with the following drugs for 30 min before agonist treatment as indicated: D1 receptor antagonist: SCH23390 (10 μM, Sigma); D2 receptor antagonist: eticlopride (20 μM, Sigma); mTORC1 inhibitor: rapamycin (100 nM, Sigma); MEK inhibitor: U0126 (20 μM); NMDAR antagonist: APV (40 μM). For each experiment, two duplicates of non-treated and antagonists treated cells were included. After incubation with the antagonists, cells were treated with SKF38393 (25 μM) for the indicated time periods. Following pharmacological treatments, cells were taken for RNA extraction, immunocytochemistry and protein synthesis detection by the surface sensing of translation (SUnSET) (Schmidt et al., 2009) method.

### Immunocytochemistry

Primary cortical cultures were fixed in cold 4% formaldehyde solution in phosphate saline buffer 0.01M (PBS) for 10 min. The cells were washed three times for 5 min with PBS+ Triton X-100 1%. The cells were then incubated for 1 h at room temperature (RT) in blocking solution of PBS + Triton X-100 1%, containing 10% fetal calf serum and 0.3% bovine serum albumin (BSA). The cells were incubated overnight at 4°C and 1 h at room temperature (RT) with the following primary antibodies diluted in the same blocking solution: phospho-eEF2 Thr56 (1:100, Cell Signaling), eEF2 (1:100, Cell Signaling), MAP2 (1:1000 Abcam), puromycin (1:1000, Millipore). Cells incubated in blocking solution lacking the primary antibody were used as negative control. After washing with PBS+1% Triton (3 × 5 min), cells were incubated for 1 h at RT with the corresponding secondary antibodies: donkey Anti-Chicken AlexaFluor^®^ 488 (1:500), donkey Anti-Rabbit and Anti-Mouse AlexaFluor^®^ 594 (1:500). The cells were then washed with PBS+1% Triton (2 × 5 min) and PBS (2 × 5 min). Finally, coverslips were mounted on Superfrost^TM^ Plus Adhesion slides (Thermo Fisher Scientific) with Slow Fade^®^ Gold antifade reagent containing DAPI (Life Technologies).

### Immunocytochemistry Quantification

Signal intensity quantification of phosphorylated eEF2 (peEF2) in the cell soma or dendrites was done by NIS Element Advanced Research (Ar) 4.5 (Nikon Japan) software in MAP2 labeled neurons. Confocal images were acquired using a Nikon 63X immersion oil objective at a resolution of 1024 × 1024 pixels. Each image was a Z series projection of 7 to 10 images, taken at depth intervals of 0.5 μm. To define the region of interest for quantification, cell bodies were identified using the DAPI nuclei stain and dendrites 10 μm away from del cell body were manually traced using NIS Element AR software on the MAP2 channel. peEF2 signal intensity (mean pixel intensity) was estimated as the peEF2 integrated fluorescence intensity divided by the area marked by the MAP2 signal.

### Puromycin Immunocytochemistry and Quantification

Cells for immunofluorescence were plated on coverslips coated with Poly-L-ornithine and laminin coating. After 14 DIV cells were pre-treated with U0126 (20 μM) or vehicle (DMSO) for 30 min and then treated with SKF38393 (25 μM) for 4 h. After SKF38393 treatment, cells were further incubated with 10 μg/ml puromycin for 10 min in the same medium and fixed in 4% paraformaldehyde. Cells were stained with anti-puromycin (1:1000, clone 4G11, EMD Millipore) and MAP2 (1:1000) antibodies, following the same procedure for peEF2Thr56 immunocytochemistry. Images were taken as Z series projection of 5 to 9 images at depth intervals of 0.25 μm at x60 magnification with an Olympus IX81 microscope using Olympus cellSens1.16 software. Quantification of puromycin incorporation was done by selecting neurons randomly in MAP2 labeled neurons and estimating the puromycin signal as mean intensity divided by the area marked by MAP2 signal using ImageJ 1.51J software. Quantification was done in a blind manner based on three independent experiments for each condition.

### SUnSET

Protein synthesis was measured by the SUnSET method. Cortical neurons were isolated and maintained for 14 days in culture, as described above. Neurons were pre-treated with U0126 (20 μM) or vehicle (DMSO) for 30 min and then treated with SKF38393 (25 μM) for 1.5 or 4 h. After SKF38393 treatment, cells were further incubated with 10 μg/ml puromycin for 10 min in the same medium. After puromycin labeling, cells were washed once with PBS and lysed in homogenization buffer (40 mM Tris–HCl, pH 8.0, 150 mM NaCl, 25 mM β-glycerophosphate, 50mM NaF, 2mM Na3VO3, 10% glycerol, 1% Triton X-100). Puromycin incorporation was detected by Western blotting using 12 D 10 antibodies for puromycin (1:5000, Millipore).

### Western Blotting

Samples in SDS sample buffer were subjected to SDS-PAGE (7.5–10%) and Western blot analysis. Lanes were loaded with an equal amount of protein. Following transferring into a nitrocellulose or PVDF membranes using *Trans-*Blot^®^ Turbo^TM^ Transfer System (Bio-Rad), bands were visualized with Ponceau staining (Bio-Rad). Membranes were blocked in 5% BSA or 5% non-fat-dry milk (depending on the primary antibody) for 1 h at RT, before being incubated overnight at 4°C with the primary antibodies: p44/42 MAP Kinase (1:5000, Cell Signaling) and Phospho-P44/42 MAP Kinase-(Thr202/Tyr204) (1:5000, Cell Signaling); S6K (1:1000, Cell Signaling), phospho-S6K(Thr389) (1:750, Cell Signaling), eEF2 (1:1000, Cell Signaling), phospho-eEF2(Thr56) (1:1000, Cell Signaling), phpho-eEF2K (Ser366) (1:1000, Cell Signaling), BDNF (1:500 Santa Cruz), synapsin 2B (1:1000 Abcam), β-Actin (1:6000, Abcam). AMPK (1:2000 Cell Signaling), phospho-AMPK (Thr172), 4E-BP (1:1,1000, Cell Signaling), phospho-4E-BP (Thr37/46; 1:1,000, Cell Signaling), p90RSK (1:2000, Cell Signaling), phospho-p90RSK (Thr573; 1:1000, Cell Signaling). Following three 5-min washing steps in Tris-buffered saline (140 mM NaCl, 20 mM Tris, pH 7.6) plus 0.1% Tween 20 (TBS-T), membranes were incubated for 1 h at room temperature with secondary HRP-linked antibodies: Goat-anti-Rabbit (IgG) HRP conjugated; Goat-anti-chicken (IgG) HRP conjugated (1:10,000, Jackson ImmunoResearch). Immunodetection was accomplished with the Enhanced Chemiluminescence EZ ECL kit (Biological Industries). Quantification of immunoblots was performed with a CCD camera and Quantity One 4.6 software (Bio-Rad). Each sample was measured relative to the background. Phosphorylation levels were calculated as the ratio of phosphorylated protein and a total amount of protein.

### RNA Extraction and qPCR of Primary Cultures

#### RNA Extraction

Tri Reagent was added directly to primary culture plates. Cells were scrapped and transferred to 1.5 ml tubes, 1-bromo, 3-chloropropane was then added at a tenth of the volume of Tri Reagent and mixed thoroughly. After phase separation by centrifugation of 15 min at 12,000 rcf, 2- propanol was added in equal volume to the RNA phase that was separated from the rest of the sample and placed in new tubes. Following 30 min of centrifugation at 12,000 rcf, the pelleted RNA was then washed once with 70% cold ethanol and centrifuged for 10 min at 7,600 rcf, the pellet was air dried and eluted in ultra pure water (Biological Industries, Beit Haemek, Israel). All reagents used were purchased from Sigma-Aldrich (Merck KGaA, Darmstadt, Germany), unless otherwise stated.

#### Reverse Transcription and qPCR

RNA samples were copied to cDNA using Applied Biosystems (Thermo Fisher, Waltham, Massachusetts, United States) High Capacity cDNA Reverse Transcription kit. The resultant cDNA was then used in TaqMan gene expression assays. The target primers used were BDNF (Mm04230607_s1), Syn2 (Mm00449780_m1), which were measured against GAPDH (Mm99999915_g1). Both reactions were carried out as per the manufacturer’s protocols. Relative quantitation was done using delta-delta ct of the target genes. One way ANOVA was carried out and *post hoc* Tukey’s test comparing each experimental group with the control was done, using GraphPad Prism software.

### Statistical Analysis

Graphs were prepared using GraphPad Prism 6.01, InStat Software (GraphPad Software, CA, United States). Data are expressed as mean ± SEM. Statistical analysis was performed using SPSS version 24. Each experiment was normalized to its own control (non-treated, NT). In each experiment, 2 replicates of NT cells were used in order to verify the cultures were homogeneous. An average of the NT samples was calculated and values obtained for other samples were divided by it. The average of the control was always calculated based on samples electrophoresed in the same gel. Each type of pharmacological experiment had 5 biological replicates (5 independent cultures). Statistical significance was determined with one-way ANOVA followed by Tukey’s *post hoc* test used for analysis of the different pharmacological manipulation experiments. Mann–Whitney analysis was used in the immunocytochemistry experiments in order to examine the differences in eEF2 phosphorylation between NT cells and SKF-treated cells in both soma and dendrites.

## Results

### D1 but Not D2 Receptor Activation Induces eEF2 Dephosphorylation Primary Neuronal Culture

The mTOR and MEK-ERK pathways are regulators of eEF2K (Redpath et al., 1993; Waelti et al., 2001; Browne et al., 2004; Proud, 2015). Activation of dopamine receptors can modulate both pathways in various types of cells, resulting in different behaviors (Nosyreva et al., 2013; Schicknick et al., 2008; Gangarossa et al., 2012; Schicknick et al., 2012; Biever et al., 2015). Therefore, we asked whether stimulation of the different dopamine receptors could induce changes in eEF2K and its sole downstream known target, eEF2 (Kenney et al., 2015).

**FIGURE 1:**
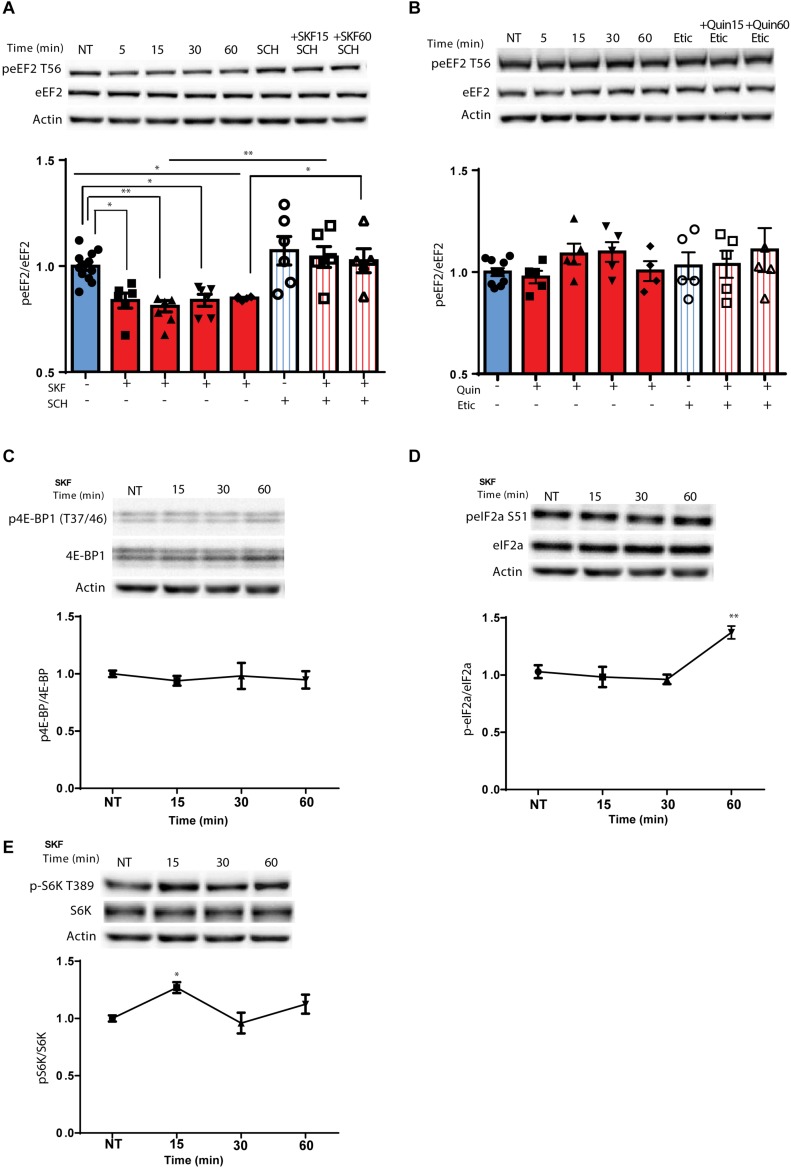
D1 but not D2 receptor enhances specific rapid eEF2 dephosphorylation with no changes in either 4E-BP or eIF2α **(A)** Cortical primary cultures from C57BL/6 mice were treated with D1 receptor agonist SKF38393 (25 μM) for the indicated time periods with or without pre-incubation with D1 receptor antagonist SCH23390 (10 μM, 30 min) followed by incubation with SKF38393. **(B)** Cortical primary cultures were treated as in panel **(A)** with D2 agonist quinpirole (10 μM) for the indicated time periods with or without pre-incubation with D2 antagonist eticlopride (20 μM, 30 min). **(C)** Representative blots and quantification of the ratios of phosphorylated 4E-BP (Thr^37/46^) to total 4E-BP from cortical primary cultures incubated with SKF38393 (25 μM) for the indicated time periods. **(D)** Representative blots and quantification of the ratios of phosphorylated eIF2a (Ser^51^) to total eIF2a from cortical primary cultures incubated with SKF38393 (25 μM) for the indicated time periods. **(E)** Representative blots and quantification of the ratios of phosphorylated S6K (Thr^389^) to total S6K from cortical primary cultures incubated with SKF38393 (25 μM) for the indicated time periods. Data are means ± SEM of five independent cultures.

Using primary cortical neuronal cultures (*n* = 6 independent cultures), we found that treatment with D1 receptor agonist SKF38393, but not the D2 agonist, quinpirole, induced rapid dephosphorylation of eEF2, which lasted for 1 h (Figure 1A; *F*(7,45) = 8.219, *p* < 0.0001, one-way ANOVA). Compared to NT: SKF 5 min, *p* = 0.02, SKF 15 min *p* = 0.006, SKF 60 min, *p* = 0.04). Pretreatment of the cells with SCH23390, a D1 receptor antagonist, blocked the D1 receptor-induced dephosphorylation of eEF2 (Figure 1A; SKF 15 min vs. SKF 15 min +SCH, *p* = 0.003; SKF 60 min vs. SKF 60 min +SCH, *p* = 0.04), while pretreatment with D2 receptor antagonist, eticlopride, did not have any effect (Figure 1B; one-way ANOVA, *F*(7,36) = 0.828, *p* = 0.57). As expected, SKF38393 treatment of primary cultures induced activation of ERK1/2 and phosphorylation of its target kinase p90RSK, which regulates the activity of eEF2K by phosphorylation on Ser366 (Dalby et al., 1998; Frodin and Gammeltoft, 1999; Wang et al., 2001; Gangarossa et al., 2012; David et al., 2014) (Supplementary Figures S1A,B). Moreover, ERK1/2 activation correlated with dephosphorylation of eEF2 by D1 receptor stimulation (*r* = −0.50, *p* < 0.05, Pearson’s correlation; Supplementary Figure S1C). Notably, eEF2 dephosphorylation at Thr^56^ correlated with eEF2K phosphorylation at Ser^366^ (Supplementary Figure S1D).

Since the mTOR cascade promotes cap-dependent translation by phosphorylation and inhibition of 4E-BP (4E-binding protein) (Santini and Klann, 2011), and D1 receptor stimulation induces phosphorylation of S6K, a well-known mTOR target (Antion et al., 2008; Hoeffer and Klann, 2010), we tested whether these mTOR downstream targets are affected by the D1 receptor. Treatment of cortical neurons with SKF38393 did not change 4E-BP phosphorylation at the same time points in which eEF2 is dephosphorylated (Figure 1C; one way ANOVA, *F*(6,30) = 0.365, *p* = 0.895), but did increase S6K phosphorylation after 15 min (Figure 1E; one way ANOVA, [*F*(3,21) = 5.513, *p* = 0.006]. Compared to NT: SKF15, *p* = 0.01, SKF30 min, *p* = 0.95, SKF60 min, *p* = 0.385). In addition, no changes were found in another translation factor examined, eukaryotic initiation factor 2 alpha (eIF2α) after short-term incubation periods. However, after 1 h eIF2α showed increased phosphorylation (Figure 1D; [*F*(3,15) = 7.787, *p* < 0.01, one-way ANOVA]. Compared to NT: SKF15, *p* = 0.98, SKF 30 min *p* = 0.86, SKF 60 min, *p* = 0.006). In addition to its negative regulators (MEK-ERK and mTOR), eEF2K also has a positive regulator which is downstream of the D1 receptor, AMP kinase (AMPK). Examining the phosphorylation of AMPK at its activation site, Thr172, revealed no difference in the phosphorylation state at the same time points when D1 receptor inhibits eEF2K at Ser366 and leads to eEF2 dephosphorylation (15 and 60 min; Supplementary Figure S1E). These results suggest that D1 activation affects the elongation phase of translation via eEF2 dephosphorylation.

### eEF2 Dephosphorylation by D1 Receptor Activation Is Dependent on the NMDA Receptor, MEK, and mTOR

**FIGURE 2:**
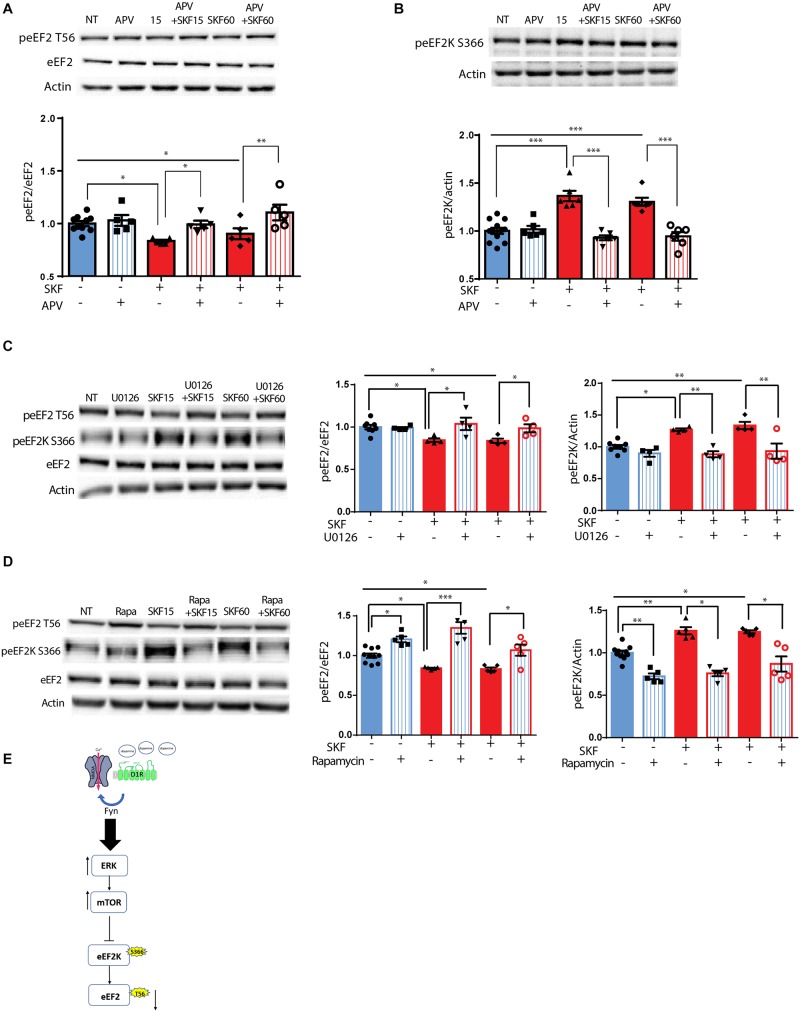
D1 receptor-dependent dephosphorylation of eEF2 requires NMDAR, MEK-ERK, and mTOR pathways. **(A)** Representative blots and quantified ratios of phosphorylated to total eEF2 and **(B)** phosphorylated eEF2K to β-actin in cortical primary cultures from C57BL/6 mice incubated with SKF38393 (25 μM) for 15 or 60 min, with or without 30 min pretreatment with NMDA receptor antagonist APV (40 μM). **(C,D)** Representative blots and quantification of the ratios of phosphorylated to total eEF2 and phosphorylated eEF2K to β-actin in cortical primary cultures incubated with SKF38393 (25 μM), for 15 or 60 min with or without pretreatment with MEK inhibitor U0126 (20 μM) for 30 min or mTOR inhibitor rapamycin (100nM) for 30 min. **(E)** Proposed model of ERK- and mTOR-dependent D1-eEF2 dephosphorylation. Data are means ± SEM of five independent cultures. ^∗^*p* < 0.05, ^∗∗^*p* < 0.001.

Following the correlation found between ERK2 activation, as indicated by its phosphorylation state, and eEF2 dephosphorylation (Supplementary Figure S1C), and since ERK is downstream of both dopamine and glutamate receptor activation (Kaphzan et al., 2006; David et al., 2014), we further asked whether the NMDA receptor plays a role in D1 receptor-dependent dephosphorylation of eEF2. To test this, primary cortical neuronal cultures were pretreated for 30 min with APV, a NMDA receptor antagonist, followed by 15 or 60 min incubation with D1 receptor agonist SKF38393. Treatment of the cells with APV prevented the dephosphorylation of eEF2 following SKF38393 treatment, both after 15 and 60 min [Figure 2A; one-way ANOVA, *F*(5,29) = 7.503, *p* < 0.001; *post hoc* test, NT vs. SKF 15 min: *p* = 0.02; NT vs. SKF 60 min: *p* = 0.02; SKF 15 min vs. SKF 15 min +APV: *p* = 0.03; SKF 60 min vs. SKF 60 min +APV: *p* = 0.001].

Since eEF2K inhibits eEF2 activity and is negatively regulated by phosphorylation on Ser^366^, we further asked whether treatment with SKF38393 increases eEF2K phosphorylation on Ser^366^ at the same time points in which we observed eEF2 dephosphorylation. Indeed, treatment of primary cultures with SKF38393 clearly induced phosphorylation of eEF2K on Ser^366^ (Figure 2B; one-way ANOVA, *F*(5,36) = 20.66, *p* < 0.001; *post hoc* test, NT vs. SKF 15 min: *p* < 0.001; NT vs. SKF 60 min: *p* < 0.001), which was blocked by pre-incubation with NMDA receptor antagonist, APV (SKF 15 min vs. SKF 15 min +APV: *p* < 0.001; SKF 60 min vs. SKF 60 min +APV: *p* < 0.001). As expected, APV pre-treatment also abolished ERK1/2 activation, consistent with previous studies from our lab (Kaphzan et al., 2006; David et al., 2014) (Supplementary Figure S2A).

Recent reports establish that inhibition of CaMKII concurrent with eEF2K-dependent increase in protein synthesis is an essential step in the manifestation of antidepressant effects of ketamine (Adaikkan et al., 2018). Since dopamine can also activate CaMKII signaling (Hasbi et al., 2009; Adaikkan et al., 2018), we examined the role of the pathway in D1 receptor-dependent eEF2 dephosphorylation. Surprisingly, treatment of cultures with SKF38393 in the presence of the CaMKII specific inhibitor TatCN21 peptide (Wong et al., 2019) demonstrated a mild contribution of CaMKII to the D1 receptor-dependent dephosphorylation of eEF2 (Supplementary Figure S2B).

Since both MEK-ERK and mTOR can lead to cross-activation and pathway convergence on substrates, as in the case of eEF2K phosphorylation (Redpath et al., 1993; Wang et al., 2001; Browne et al., 2004; Lenz and Avruch, 2005), we sought to differentiate between these pathways, and test whether one of them is more dominant in the inhibition of eEF2K and in inducing D1 receptor-dependent eEF2 dephosphorylation in neurons. Pre-incubation of cortical neurons (5 independent cultures) with the U0126 compound, a MEK inhibitor, blocked the phosphorylation of eEF2K at Ser^366^ (Figure 2C; one-way ANOVA *F*(5,22) = 10.88, *p* < 0.0001; *Post hoc* test, NT vs. SKF 15 min: *p* = 0.01; NT vs. SKF 60 min: *p* = 0.002; SKF 15 min vs. SKF 15 min +U0126: *p* = 0.002; SKF 60 min vs. SKF 60 min +U0126: *p* = 0.001), and inhibited eEF2 dephosphorylation (Figure 2C; One-way ANOVA *F*(5,22) = 4.60, *p* = 0.005; *Post hoc* test, NT vs SKF 15 min: *p* = 0.04; NT vs. SKF 60 min: *p* = 0.03; SKF 15 min vs. SKF 15 min +U0126: *p* = 0.03; SKF 60 min vs. SKF 60 min +U0126: *p* = 0.04). As expected, U0126 blocked ERK activation (Supplementary Figure S2A). Interestingly, U0126 also blocked S6K phosphorylation as reported previously (Kenney et al., 2015, Supplementary Figure S2C). Pre-incubation of cortical neurons with the mTOR inhibitor, rapamycin, enhanced the phosphorylation of eEF2 (Figure 2D; one-way ANOVA *F*(5,29) = 19.34, *p* < 0.0001; *post hoc* test, NT vs. rapa: *p* = 0.01; NT vs. SKF 15 min: *p* = 0.03; NT vs. SKF 15 min +rapa: *p* < 0.001; NT vs. SKF 60 min: *p* = 0.04; SKF 15 min vs. SKF 15 min +rapa: *p* < 0.0001; SKF 60 min vs. SKF 60 min +rapa: *p* = 0.01) and reduced eEF2K at Ser^366^ (Figure 2D; one-way ANOVA *F*(5,29) = 26.161, *p* < 0.0001; *post hoc* test, NT vs. rapa: *p* < 0.001; NT vs. SKF 15 min: *p* = 0.001; NT vs. SKF 60 min: *p* = 0.02; SKF 15 min vs. SKF 15 min +rapa: *p* < 0.001; SKF 60 min vs. SKF 60 min +rapa: *p* < 0.001).

Interestingly, incubation of the cells with rapamycin alone resulted in enhancement of eEF2 phosphorylation (Supplementary Figure S2D). As expected, rapamycin alone blocked S6K phosphorylation as well as the D1-dependent phosphorylation after 15 min of incubation. However, no effect was found on ERK (Supplementary Figure S2D). These findings imply that the MEK-ERK pathway is upstream of the mTOR pathway following D1 receptor stimulation (Figure 2E).

### Dopamine D1 Receptor Dephosphorylates eEF2^Thr56^ in Neuronal Dendrites and Somata

**FIGURE 3:**
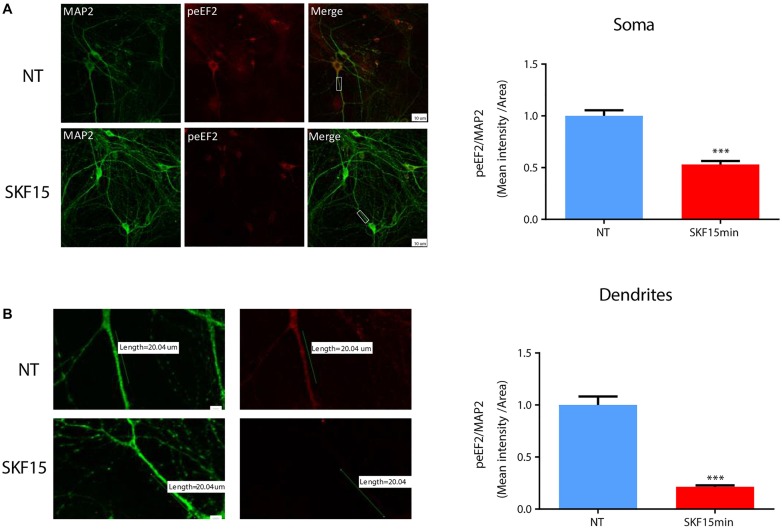
D1 receptor activation induces eEF2 dephosphorylation in dendrites more than in cell soma. **(A)** Immunofluorescence and mean intensity quantification of phospho-eEF2 (Thr^56^, red) in neuronal somata mean intensity in primary cortical cultures from C57BL/6 mice were treated with SKF38393 (25 μM) for 15 min. Scale bar, 20 μm. Images represent 15 to 20 neurons (labeled with MAP2, green) from four independent cultures. **(B)** Immunofluorescence and mean intensity quantification of phospho-eEF2 (red) in dendrites in primary cortical cultures treated with SKF38393 (25 μM) for 15 min. Scale bar, 10 μm. Images represent 15 to 20 neurons (labeled with MAP2, green) from four independent cultures. Means ± SEM are shown in all graphs. ^∗∗∗^*p* < 0.0001.

Regulation of eEF2K activity and reduction of eEF2 phosphorylation by synaptic receptors such as the NMDA receptor serves as one possible way to translate mRNA in dendrites (Autry et al., 2011). To examine whether dopaminergic transmission can also regulate dendritic eEF2 phosphorylation, we performed immunocytochemical analysis of cortical neurons from wild-type mice (*n* = 5 independent cultures) treated with SKF38393 for 15 min. The results revealed a reduction in phospho-eEF2 immunoreactivity in most of the neurons analyzed. The effect was seen in both neuronal soma (Figure 3A; *U* = 8839, *Z* = −8.994, *p* < 0.0001; Mann–Whitney test) and dendrites (Figure 3B; *U* = 3975, *Z* = −6.489, *p* < 0.0001; Mann–Whitney test). Phospho-eEF2 antibody specificity was tested in primary cultures derived from eEF2K-KO mice. No immunoreactivity was detected in cortical neurons from eEF2K-KO cultures (Supplementary Figure S3). Moreover, although our cultures are mixed, containing both neurons and glia, the effect of D1 receptor activation on eEF2 occurred specifically in neurons, since no changes were found in glia cells (Supplementary Figure S4). These results provide evidence that D1 receptor activation reduces eEF2 phosphorylation in both dendrites and soma.

### Dopamine D1 Receptor Activation Enhances Protein Synthesis in Cultured Cortical Neurons

**FIGURE 4:**
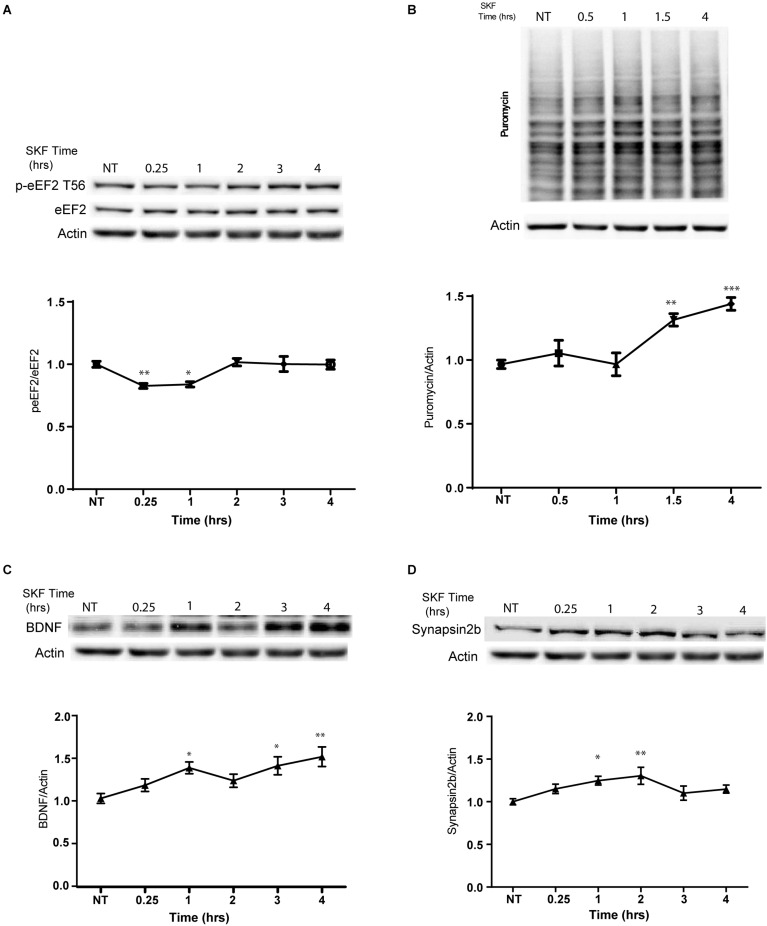
D1 receptor activation promotes general protein synthesis. **(A)** Western blot analysis of eEF2 (Thr^56^) phosphorylation in cortical primary cultures treated with SKF38393 (25 μM) for the indicated time periods. **(B)** Time-dependent increase in protein synthesis in primary cultures from C57BL/6 mice treated with SKF38393 (25 μM) for the indicated time periods. Puromycin signal was quantified and normalized to β-actin loading control. **(C)** Representative Western blots and quantification of BDNF and synapsin2b **(D)** following prolonged treatment with SKF38393 (25 μM). Means ± SEM are shown in all graphs. ^∗^*p* < 0.05, ^∗∗^*p* < 0.001.

In light of our immunocytochemistry results, we further asked whether the dopamine D1 receptor-dependent eEF2 dephosphorylation coincides with enhanced protein synthesis. Treatment with SKF38393 resulted in rapid de-phosphorylation of eEF2 which returned to baseline after 2 h (Figure 4A; one way ANOVA *F*(5,29) = 6.338, *p* < 0.0001. *Post hoc* test, NT vs. SKF 15 min: *p* = 0.006; NT vs. SKF 60 min: *p* = 0.01; SKF 15 min vs. SKF2 h: *p* = 0.001; SKF 15 min vs. 3 h: *p* = 0.02; SKF 15 min vs. 4 h: *p* = 0.02; SKF 60 min vs. SKF2 h: *p* = 0.01; SKF60 vs. SKF3 h: *p* = 0.03; SKF 60 min vs. SKF4 h: *p* = 0.04. We utilized SUnSET, a non-radioactive method of monitoring global protein synthesis in cultured cells that uses puromycin to tag nascent proteins (Schmidt et al., 2009). Time-course experiments showed that puromycin incorporation was significantly increased only after 1.5 and 4 h of incubation with SKF38393 (Figure 4B; One way ANOVA *F*(4,31) = 11.89, *p* < 0.001. *Post hoc* test, NT vs. SKF1.5 h: *p* = 0.002; NT vs. SKF4 h: *p* < 0.001). Similar increase in protein synthesis was reported after ketamine administration, which leads to a reduction in phosphorylation levels of eEF2 via the inhibition of its kinase and CaMKII (Adaikkan et al., 2018).

We further examined whether SKF38393 stimulation causes rapid upregulation of specific proteins related to the eEF2K pathway. Indeed, we observed increased BDNF (Autry et al., 2011; Nosyreva et al., 2013) and synapsin 2B (Heise et al., 2017) protein but not mRNA expression (Figures 4C,D). A mild increase in the levels of both proteins was observed after 15 min and reached significance following 1 h of incubation with SKF38393 (Figures 4C,D; BDNF: one way ANOVA *F*(2,37) = 5.354, *p* = 0.009. *Post hoc*: NT vs. SKF1.5 h, *p* = 0.04; NT vs. SKF4 h, *p* = 0.01; Synapsin2b: One way ANOVA *F*(5,29) = 3.744, *p* = 0.01. *Post hoc*: NT vs. SKF1 h, *p* = 0.04; NT vs. SKF2 h, *p* = 0.0.008). This increase lasted 4 h without affecting their mRNA levels (BDNF: One way ANOVA *F*(5,16) = 1.991, *p* = 0.134; synapsin 2b: *F*(5,18) = 0.567, *p* = 0.723). No changes in puromycin incorporation were found following incubation with the D2 receptor agonist quinpirole (Supplementary Figure S5A).

### NMDAR, MEK, and eEF2K Are Necessary for D1-Dependent Increased Protein Synthesis

**FIGURE 5:**
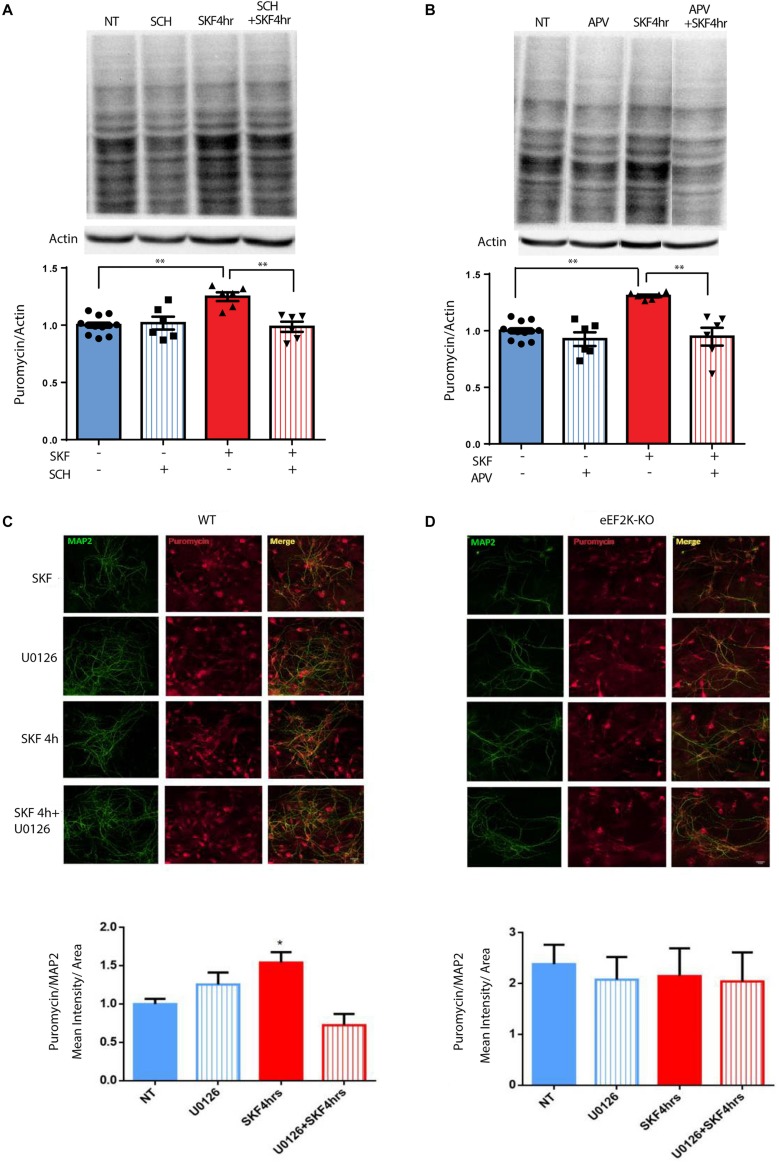
NMDA receptor, MEK/ERK, and eEF2 are necessary for D1 receptor-dependent increase in protein synthesis. **(A)** Time-dependent increase in protein synthesis in primary cultures from C57BL/6 mice treated with SKF38393 (25 μM) with/without SCH23390 for 4 h. **(B)** Time-dependent increase in protein synthesis in primary cultures from C57BL/6 mice treated with SKF38393 (25 μM) with/without APV (40 μM) for 4 h. **(C,D)** Cortical primary cultures from eEF2K-KO mice or their littermates wild types were incubated with SKF38393 (25 μM) for 4 h with or without pre-incubation with MEK inhibitor U0126 (20 μM) for 30 min, followed by incubation with puromycin (1 μg/ml) for 10 min. Puromycin was detected by immunofluorescence (red), and quantified by measuring its mean intensity in MAP2 (green) positive cells. Means ± SEM are shown in all graphs, *p < 0.05, ^∗∗^*p* < 0.001.

The results presented in Figure 1 and Supplementary Figure S5A demonstrate that D1 but not D2 receptor activation can lead to eEF2 dephosphorylation. blocking D1 receptor with its antagonist SCH23390 decreased puromycin labeling, suggesting D1 but not D2 receptor dependence (Figure 5A; one-way ANOVA *F*(3,26) = 9.75, *p* < 0.001; *post hoc* test, NT vs. SCH: *p* = 0.98; NT vs. SKF 4 h: *p* < 0.001; NT vs. SKF 4 h +SCH: *p* = 0.99; SKF 4 h vs. SKF 4 h +SCH: *p* = 0.001]. Our results also imply the involvement of the NMDA receptor in D1-dependent eEF2 dephosphorylation. In order to examine the necessity of the NMDA receptor for D1 receptor-induced protein synthesis, we measured puromycin incorporation in the presence of NMDA receptor antagonist APV after 4 h of incubation at the time point of a large increase in protein synthesis (Figure 5B, Supplementary Figure S5A). SKF38393 treatment with NMDA receptor blockade abolished the induction of protein synthesis, suggesting a pivotal role for the NMDA receptor in the D1-dependent protein synthesis (Figure 5B; one-way ANOVA *F*(3,26) = 13.67, *p* < 0.0001; *post hoc* test, NT vs. APV: *p* = 0.60; NT vs. SKF 4 h: *p* < 0.001; SKF4 h vs. SKF 4 h +APV: *p* < 0.001].

To test if the eEF2K/eEF2 pathway is necessary for the D1 receptor increase of protein synthesis, we used primary cultures from eEF2K-KO mice. These mice show no eEF2 phosphorylation, but display normal phosphorylation of other translation factors (Heise et al., 2017; Adaikkan et al., 2018). Cortical cultures from eEF2K-KO mice and their wild-type littermates were treated for 4 h with SKF38393 and global protein synthesis was analyzed by SUnSET. Immunocytochemistry and western blot analyses of puromycin incorporation showed a significant increase in wild type but not in eEF2K-KO cultures (*n* = 4 independent cultures) following SKF38393 incubation (Figures 5C,D, Supplementary Figures S5B,C). To probe whether the D1 receptor activation-induced increase in translation is dependent on ERK/mTOR/eEF2K/eEF2 signaling, we pre-treated cortical neurons derived from wild type and eEF2-KO mice with or without MEK inhibitor U0126, followed by SKF38393 treatment. We found that the SKF38393-mediated increase in translation was reduced by treatment with U0126 in wild type neurons, while no change in puromycin incorporation was detected in eEF2K-KO mouse-derived cultures treated with U0126 (Figures 5C,D and Supplementary Figures S5B,C; one-way ANOVA *F*(3,17) = 7.909, *p* = 0.002; *post hoc* test, NT vs. SKF 4 h: *p* = 0.01; SKF 4 h vs. SKF 4 h +U0126: *p* = 0.001). These results suggest that the MEK/ERK and eEF2 pathways are necessary for the D1 receptor-dependent increase in protein synthesis in cortical neurons.

## Discussion

In summary, while previous studies have shown that dopamine regulates mRNA translation (Smith et al., 2005), our data show for the first time that this regulation is mediated by the eEF2 pathway. Specifically, we show that dopamine D1 receptor activation in neurons inhibits eEF2K, resulting in reduced eEF2 phosphorylation. Furthermore, we observed a small but significant increase in general protein synthesis in neurons 1 h after D1 activation with increase of specific eEF2K-related proteins such as BDNF and synapsin 2b. The eEF2 rapid de-phosphorylation following D1 activation, occurred at the same time with S6K activation, but there was no phosphorylation changes at these time points in other translation factors as 4E-BP or eIF2α. Both dopamine and eIF2α pathway are pivotal for memory consolidation (Belelovsky et al., 2009; Stern et al., 2013; Ounallah-Saad et al., 2014; Segev et al., 2016; Gal-Ben-Ari et al., 2018; Sharma et al., 2018), however, possibly via different molecular and cellular mechanisms. From a signal transduction perspective, NMDA receptor is required for the D1 receptor-dependent induction of MEK/mTOR activity that leads to inactivation of eEF2K.

Our data support the view that dopamine D1 receptor activation regulates neuronal proteostasis, specifically affecting the elongation phase of translation, which mediates the effect of antidepressants in the CNS (Flight, 2011; Adaikkan et al., 2018; Hare et al., 2019). D1 receptor activation has different effects including enhanced learning and memory in various learning paradigms such as CTA, fear conditioning, and object recognition in rodents (Nagai et al., 2007; Nosyreva et al., 2013; Balderas et al., 2013; David et al., 2014; Péczely et al., 2014a, b). Infusion of D1 receptor antagonist SCH23390 into the prefrontal cortex of monkeys or rats impaired spatial working memory, while D2 receptor antagonist showed no effect.

Interestingly, the time frame of eEF2 dephosphorylation after D1 receptor activation proceed the time of increased general protein synthesis. However, they are linked, most probably indirectly, since no increase in puromycin incorporation was detected in the eEF2K-KO mice cultures.

Our data provide further evidence of the well-known relationship between D1 and NMDA receptors and its effect on signal transduction (Dunah and Standaert, 2001; Lee et al., 2002; Pei et al., 2004; Martina and Bergeron, 2008; Stramiello and Wagner, 2008; David et al., 2014). The results indicate that, in addition to the canonical calcium-calmodulin-dependent activation of eEF2K following NMDA receptor stimulation, NMDAR-D1 interaction induces translational changes via ERK and S6K signaling cascades. Moreover, the eEF2K pathway accounts for the increase in protein synthesis following dopamine D1 receptor activation.

Although eEF2K is activated by the Ca^2+^-Calmodulin complex, it is also regulated by phosphorylation. mTOR- and MEK-dependent phosphorylation of eEF2K reduces its activity, while PKA- and AMPK-mediated phosphorylation does the opposite (Wang et al., 2001; Heise et al., 2014; Tan et al., 2014). Given this complex regulation of its function, we propose that eEF2K functions as a pivotal convergence signaling hub, linking synaptic information to regulation of specific protein synthesis. In addition, it was suggested that eEF2 acts as a biochemical sensor to discriminate between evoked action potential and spontaneous miniature synaptic transmission (Sutton et al., 2007).

**FIGURE 6:**
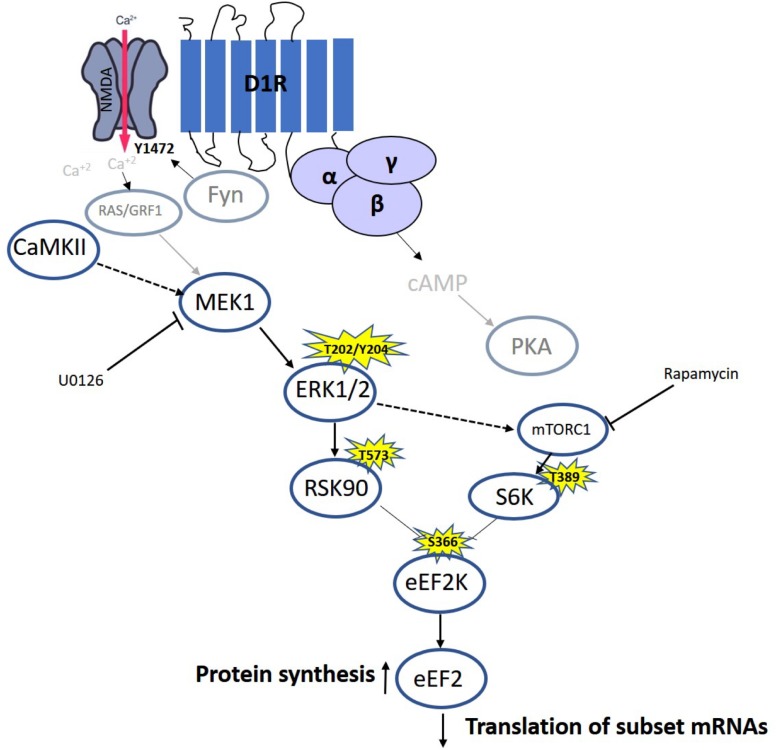
Model of D1 receptor-dependent dephosphorylation of eEF2 in cortical neurons. Dopamine D1 receptors activated in dendrites lead to small calcium influx via the NMDA receptor, which elevates both MEK–ERK and mTOR pathways. Both pathways inhibit eEF2K activity by phosphorylating it on Ser366, leading to eEF2 Thr56 dephosphorylation and increased protein synthesis.

Our results and previous time-dependent studies suggest the existence of different phases in eEF2 regulation in neurons: The first phase causes a rapid increase in eEF2 phosphorylation, due to synaptic activation and NMDA receptor-dependent high calcium influx (Belelovsky et al., 2009; Gildish et al., 2012; Taha et al., 2013), which leads to inhibition of general protein synthesis and an increase in specific proteins such as c-Fos, Arc (Chotiner et al., 2003; Park et al., 2008), and CaMKII (Scheetz et al., 2000). The second phase is mediated by dopamine D1 receptor-dependent eEF2 dephosphorylation and the molecular pathway described in the study, which accumulates with increased expression of a specific subset of proteins such as BDNF (Figure 6). The third phase with prolong increase in protein synthesis (between 1.5and 4 h) occurs long after eEF2 reduced phsophorylation is back to baseline, most probably in an indirect way.

In a similar manner, certain levels of dopamine D1 receptor stimulation in dendrites of specific neurons can gate out “noise”, while high levels, e.g. during stress (Arnsten et al., 2015; Hare et al., 2019), suppress delayed firing. For instance, maintenance of synaptic strength in hippocampal slices treated with low concentrations of dopamine D1 agonist SKF 38393 requires MEK and CaMKII activation, while in slices treated with high concentrations, maintenance of synaptic strength is dependent only on MEK activation (Barcomb et al., 2016). The authors reported that the increase in dopamine levels in the mPFC following ketamine administration increases D1R signaling and contributes to the synaptic actions of ketamine. Our results suggest a mild contribution of CaMKII to eEF2 dephosphorylation following D1 receptor stimulation. This mild dependency could be part of the mechanism underlying the effect of ketamine on CaMKII and eEF2K, resulting in increased protein synthesis and induction of its rapid antidepressant effect (Adaikkan et al., 2018; Chang et al., 2019; Hare et al., 2019). Further investigation will be needed in order to establish a direct correlation between dopamine- and ketamine-dependent activation of CaMKII. Nonetheless, assuming eEF2 is a main target for D1 activation, it is not surprising that the dopamine D1 receptor agonist, in a similar way to ketamine, can potentially serve as an antidepressant (D’Aquila et al., 1994).

Our findings establish a link between dopamine D1 receptor activation and eEF2K activity, opening a door to better understanding the molecular mechanism underlying the effect of antidepressants and role of neuromodulators in synaptic plasticity, addiction, and memory formation. Future studies aiming to better understand neurodegenerative diseases and depression-like syndrome, combined with a circuit approach and behavioral paradigms will better link the eEF2 pathway and dopamine to establish the eEF2 pathway as a potential target for therapy.

## Data Availability Statement

The datasets generated for this study are available on request to the corresponding author.

## Ethics Statement

The animal study was reviewed and approved by the Institutional Animal Care and Use Committee of the University of Haifa.

## Author Contributions

OD and IB designed and performed the experiments, analyzed the data, and wrote the manuscript. NG contributed data to mRNA analysis. SG-B-A contributed to the primary culture preparations and edited the manuscript. KR designed experiments, supervised the project, and wrote the manuscript.

## Conflict of Interest

The authors declare that the research was conducted in the absence of any commercial or financial relationships that could be construed as a potential conflict of interest.

## References

[B1] Adaikkan C. Taha E. Barrera I. David O. Rosenblum K. (2018). Calcium/Calmodulin-Dependent Protein Kinase II and Eukaryotic Elongation Factor 2 Kinase Pathways Mediate the Antidepressant Action of Ketamine. *Biol. Psychiatry* 84 65–75. 10.1016/j.biopsych.2017.11.028 29395043

[B2] Antion M. D. Merhav M. Hoeffer C. A. Reis G. Kozma S. C. Thomas G. (2008). Removal of S6K1 and S6K2 leads to divergent alterations in learning, memory, and synaptic plasticity. *Learn. Mem.* 15 29–38. 10.1101/lm.661908 18174371 PMC2170513

[B3] Arnsten A. F. Wang M. Paspalas C. D. (2015). Dopamine’s actions in primate prefrontal cortex: challenges for treating cognitive disorders. *Pharmacol. Rev.* 67 681–696. 10.1124/pr.115.010512 26106146 PMC4485014

[B4] Autry A. E. Adachi M. Nosyreva E. Na E. S. Los M. F. Cheng P.-F. (2011). NMDA receptor blockade at rest triggers rapid behavioural antidepressant responses. *Nature* 475 91–95. 10.1038/nature10130 21677641 PMC3172695

[B5] Balderas I. Moreno-Castilla P. Bermudez-Rattoni F. (2013). Dopamine D1 receptor activity modulates object recognition memory consolidation in the perirhinal cortex but not in the hippocampus. *Hippocampus* 23 873–878. 10.1002/hipo.22143 23674387

[B6] Barcomb K. Hell J. W. Benke T. A. Bayer K. U. (2016). The CaMKII/GluN2B protein interaction maintains synaptic strength. *J. Biol. Chem.* 291 16082–16089. 10.1074/jbc.M116.734822 27246855 PMC4965558

[B7] Barrera I. Hernandez-Kelly L. C. Castelan F. Ortega A. (2008). Glutamate-dependent elongation factor-2 phosphorylation in Bergmann glial cells. *Neurochem. Int.* 52 1167–1175. 10.1016/j.neuint.2007.12.006 18222016

[B8] Belelovsky K. Kaphzan H. Elkobi A. Rosenblum K. (2009). Biphasic activation of the mTOR pathway in the gustatory cortex is correlated with and necessary for taste learning. *J. Neurosci.* 29 7424–7431. 10.1523/JNEUROSCI.3809-08.2009 19515910 PMC6665417

[B9] Biever A. Puighermanal E. Nishi A. David A. Panciatici C. Longueville S. (2015). PKA-dependent phosphorylation of ribosomal protein S6 does not correlate with translation efficiency in striatonigral and striatopallidal medium-sized spiny neurons. *J. Neurosci.* 35 4113–4130. 10.1523/JNEUROSCI.3288-14.2015 25762659 PMC6605295

[B10] Boyd K. N. Mailman R. B. (2012). Dopamine receptor signaling and current and future antipsychotic drugs. *Handb. Exp. Pharmacol.* 2012 53–86. 10.1007/978-3-642-25761-2_3 23129328 PMC4711768

[B11] Browne G. J. Finn S. G. Proud C. G. (2004). Stimulation of the AMP-activated protein kinase leads to activation of eukaryotic elongation factor 2 kinase and to its phosphorylation at a novel site, serine 398. *J. Biol. Chem.* 279 12220–12231. 10.1074/jbc.M309773200 14709557

[B12] Chang L. Zhang K. Pu Y. Qu Y. Wang S. M. Xiong Z. (2019). Lack of dopamine D1 receptors in the antidepressant actions of (R)-ketamine in a chronic social defeat stress model. *Eur. Arch. Psychiatry Clin. Neurosci.* 270 271–275. 10.1007/s00406-019-01012-1011 30927075

[B13] Chotiner J. K. Khorasani H. Nairn A. C. O’dell T. J. Watson J. B. (2003). Adenylyl cyclase-dependent form of chemical long-term potentiation triggers translational regulation at the elongation step. *Neuroscience* 116 743–752. 10.1016/s0306-4522(02)00797-792 12573716

[B14] Dalby K. N. Morrice N. Caudwell F. B. Avruch J. Cohen P. (1998). Identification of regulatory phosphorylation sites in mitogen-activated protein kinase (MAPK)-activated protein kinase-1a/p90rsk that are inducible by MAPK. *J. Biol. Chem.* 273 1496–1505. 10.1074/jbc.273.3.1496 9430688

[B15] D’Aquila P. S. Brain P. Willner P. (1994). Effects of chronic mild stress on performance in behavioural tests relevant to anxiety and depression. *Physiol. Behav.* 56 861–867. 10.1016/0031-9384(94)90316-6 7824585

[B16] David O. Barrera I. Chinnakkaruppan A. Kaphzan H. Nakazawa T. Yamamoto T. (2014). Dopamine-induced tyrosine phosphorylation of NR2B (Tyr1472) is essential for ERK1/2 activation and processing of novel taste information. *Front. Mol. Neurosci.* 7:66. 10.3389/fnmol.2014.00066 25100942 PMC4103512

[B17] David O. Barrera I. Koltun B. Ironi S. Gal-Ben-Ari S. Rosenblum K. (2018). MEK/mTOR-dependent D1 dopamine receptor activation induces local protein synthesis via eEF2 dephosphorylation in neurons. *bioRxiv* [Preprint]. 10.1101/447714

[B18] Duman R. S. Voleti B. (2012). Signaling pathways underlying the pathophysiology and treatment of depression: novel mechanisms for rapid-acting agents. *Trends Neurosci.* 35 47–56. 10.1016/j.tins.2011.11.004 22217452 PMC3278537

[B19] Dunah A. W. Standaert D. G. (2001). Dopamine D1 receptor-dependent trafficking of striatal NMDA glutamate receptors to the postsynaptic membrane. *J. Neurosci.* 21 5546–5558. 10.1523/JNEUROSCI.21-15-05546.2001 11466426 PMC6762635

[B20] Flight M. H. (2011). Mood disorders: targeting protein synthesis for fast antidepressant action. *Nat. Rev. Drug. Discov.* 10:577. 10.1038/nrd3520 21804593

[B21] Frodin M. Gammeltoft S. (1999). Role and regulation of 90 kDa ribosomal S6 kinase (RSK) in signal transduction. *Mol. Cell. Endocrinol.* 151 65–77. 10.1016/s0303-7207(99)00061-1 10411321

[B22] Gal-Ben-Ari S. Barrera I. Ehrlich M. Rosenblum K. (2018). PKR: a kinase to remember. *Front. Mol. Neurosci.* 11:480. 10.3389/fnmol.2018.00480 30686999 PMC6333748

[B23] Gangarossa G. Longueville S. De Bundel D. Perroy J. Herve D. Girault J. A. (2012). Characterization of dopamine D1 and D2 receptor-expressing neurons in the mouse hippocampus. *Hippocampus* 22 2199–2207. 10.1002/hipo.22044 22777829

[B24] Gildish I. Manor D. David O. Sharma V. Williams D. Agarwala U. (2012). Impaired associative taste learning and abnormal brain activation in kinase-defective eEF2K mice. *Learn. Mem.* 19 116–125. 10.1101/lm.023937.111 22366775 PMC3293518

[B25] Hare B. D. Ghosal S. Duman R. S. (2017). Rapid acting antidepressants in chronic stress models: molecular and cellular mechanisms. *Chronic. Stress* 1. 10.1177/2470547017697317 28649673 PMC5482287

[B26] Hare B. D. Shinohara R. Liu R. J. Pothula S. Dileone R. J. Duman R. S. (2019). Optogenetic stimulation of medial prefrontal cortex Drd1 neurons produces rapid and long-lasting antidepressant effects. *Nat. Commun.* 10:223. 10.1038/s41467-018-08168-8169 30644390 PMC6333924

[B27] Hasbi A. Fan T. Alijaniaram M. Nguyen T. Perreault M. L. O’Dowd B. F. (2009). Calcium signaling cascade links dopamine D1-D2 receptor heteromer to striatal BDNF production and neuronal growth. *Proc. Natl. Acad. Sci. U.S.A.* 106 21377–21382. 10.1073/pnas.0903676106 19948956 PMC2795506

[B28] Heise C. Gardoni F. Culotta L. Di Luca M. Verpelli C. Sala C. (2014). Elongation factor-2 phosphorylation in dendrites and the regulation of dendritic mRNA translation in neurons. *Front. Cell. Neurosci.* 8:35. 10.3389/fncel.2014.00035 24574971 PMC3918593

[B29] Heise C. Taha E. Murru L. Ponzoni L. Cattaneo A. Guarnieri F. C. (2017). eEF2K/eEF2 pathway controls the excitation/inhibition balance and susceptibility to epileptic seizures. *Cereb. Cortex* 27 2226–2248. 10.1093/cercor/bhw075 27005990 PMC5963824

[B30] Hoeffer C. A. Klann E. (2010). mTOR signaling: at the crossroads of plasticity, memory and disease. *Trends Neurosci.* 33 67–75. 10.1016/j.tins.2009.11.003 19963289 PMC2821969

[B31] Im H. I. Nakajima A. Gong B. Xiong X. Mamiya T. Gershon E. S. (2009). Post-training dephosphorylation of eEF-2 promotes protein synthesis for memory consolidation. *PLoS One* 4:e7424. 10.1371/journal.pone.0007424 19823585 PMC2757674

[B32] Kaphzan H. O’riordan K. J. Mangan K. P. Levenson J. M. Rosenblum K. (2006). NMDA and dopamine converge on the NMDA-receptor to induce ERK activation and synaptic depression in mature hippocampus. *PLoS One* 1:e138. 10.1371/journal.pone.0000138 17205142 PMC1762427

[B33] Kenney J. W. Sorokina O. Genheden M. Sorokin A. Armstrong J. D. Christopher X. (2015). Dynamics of elongation factor 2 kinase regulation in cortical neurons in response to synaptic activity. *J. Neurosci.* 35 3034–3047. 10.1523/JNEUROSCI.2866-14.2015 25698741 PMC4331626

[B34] Lee F. J. Xue S. Pei L. Vukusic B. Chery N. Wang Y. (2002). Dual regulation of NMDA receptor functions by direct protein-protein interactions with the dopamine D1 receptor. *Cell* 111 219–230. 10.1016/s0092-8674(02)00962-5 12408866

[B35] Lenz G. Avruch J. (2005). Glutamatergic regulation of the p70S6 kinase in primary mouse neurons. *J. Biol. Chem.* 280 38121–38124. 10.1074/jbc.C500363200 16183639

[B36] Martina M. Bergeron R. (2008). D1 and D4 dopaminergic receptor interplay mediates coincident G protein-independent and dependent regulation of glutamate NMDA receptors in the lateral amygdala. *J. Neurochem.* 106 2421–2435. 10.1111/j.1471-4159.2008.05584.x 18662324

[B37] Montanaro L. Sperti S. Testoni G. Mattioli A. (1976). Effect of elongation factor 2 and of adenosine diphosphate-ribosylated elongation factor 2 on translocation. *Biochem. J.* 156 15–23. 10.1042/bj1560015 182140 PMC1163712

[B38] Nagai T. Takuma K. Kamei H. Ito Y. Nakamichi N. Ibi D. (2007). Dopamine D1 receptors regulate protein synthesis-dependent long-term recognition memory via extracellular signal-regulated kinase 1/2 in the prefrontal cortex. *Learn. Mem.* 14 117–125. 10.1101/lm.461407 17337702 PMC1838552

[B39] Nosyreva E. Szabla K. Autry A. E. Ryazanov A. G. Monteggia L. M. Kavalali E. T. (2013). Acute suppression of spontaneous neurotransmission drives synaptic potentiation. *J. Neurosci.* 33 6990–7002. 10.1523/JNEUROSCI.4998-12.2013 23595756 PMC3661220

[B40] Ounallah-Saad H. Sharma V. Edry E. Rosenblum K. (2014). Genetic or pharmacological reduction of PERK enhances cortical-dependent taste learning. *J. Neurosci.* 34 14624–14632. 10.1523/JNEUROSCI.2117-14.2014 25355215 PMC6608429

[B41] Park S. Park J. M. Kim S. Kim J. A. Shepherd J. D. Smith-Hicks C. L. (2008). Elongation factor 2 and fragile X mental retardation protein control the dynamic translation of Arc/Arg3.1 essential for mGluR-LTD. *Neuron* 59 70–83. 10.1016/j.neuron.2008.05.023 18614030 PMC2743934

[B42] Péczely L. Ollmann T. László K. Kovács A. Gálosi R. Szabá A. (2014a). Effects of ventral pallidal D1 dopamine receptor activation on memory consolidation in morris water maze test. *Behav. Brain. Res.* 274 211–218. 10.1016/j.bbr.2014.07.031 25138696

[B43] Péczely L. Ollmann T. László K. Kovács A. Gálosi R. Szabá A. (2014b). Role of D1 dopamine receptors of the ventral pallidum in inhibitory avoidance learning. *Behav. Brain. Res.* 270 131–136. 10.1016/j.bbr.2014.04.054 24815313

[B44] Pei L. Lee F. J. Moszczynska A. Vukusic B. Liu F. (2004). Regulation of dopamine D1 receptor function by physical interaction with the NMDA receptors. *J. Neurosci.* 24 1149–1158. 10.1523/JNEUROSCI.3922-03.2004 14762133 PMC6793575

[B45] Proud C. G. (2015). Regulation and roles of elongation factor 2 kinase. *Biochem. Soc. Trans.* 43 328–332. 10.1042/BST20140323 26009171

[B46] Redpath N. T. Price N. T. Severinov K. V. Proud C. G. (1993). Regulation of elongation factor-2 by multisite phosphorylation. *Eur. J. Biochem.* 213 689–699. 10.1111/j.1432-1033.1993.tb17809.x 8386634

[B47] Salamone J. D. Correa M. Mingote S. Weber S. M. (2003). Nucleus accumbens dopamine and the regulation of effort in food-seeking behavior: implications for studies of natural motivation, psychiatry, and drug abuse. *J. Pharmacol. Exp. Ther.* 305 1–8. 10.1124/jpet.102.035063 12649346

[B48] Santini E. Klann E. (2011). Dysregulated mTORC1-Dependent translational control: from brain disorders to psychoactive drugs. *Front. Behav. Neurosci.* 5:76. 10.3389/fnbeh.2011.00076 22073033 PMC3210466

[B49] Scheetz A. J. Nairn A. C. Constantine-Paton M. (2000). NMDA receptor-mediated control of protein synthesis at developing synapses. *Nat. Neurosci.* 3 211–216. 10.1038/72915 10700251

[B50] Schicknick H. Reichenbach N. Smalla K. H. Scheich H. Gundelfinger E. D. Tischmeyer W. (2012). Dopamine modulates memory consolidation of discrimination learning in the auditory cortex. *Eur. J. Neurosci.* 35 763–774. 10.1111/j.1460-9568.2012.07994.x 22339853

[B51] Schicknick H. Schott B. H. Budinger E. Smalla K. H. Riedel A. Seidenbecher C. I. (2008). Dopaminergic modulation of auditory cortex-dependent memory consolidation through mTOR. *Cereb. Cortex* 18 2646–2658. 10.1093/cercor/bhn026 18321872 PMC2567422

[B52] Schmidt E. K. Clavarino G. Ceppi M. Pierre P. (2009). SUnSET, a nonradioactive method to monitor protein synthesis. *Nat. Methods* 6 275–277. 10.1038/nmeth.1314 19305406

[B53] Schultz W. (1998). The phasic reward signal of primate dopamine neurons. *Adv. Pharmacol.* 42 686–690. 10.1016/s1054-3589(08)60841-8 9327992

[B54] Segev Y. Livne A. Mints M. Rosenblum K. (2016). Concurrence of high fat diet and APOE gene induces allele specific metabolic and mental stress changes in a mouse model of Alzheimer’s disease. *Front. Behav. Neurosci.* 10:170. 10.3389/fnbeh.2016.00170 27656136 PMC5011130

[B55] Sharma V. Ounallah-Saad H. Chakraborty D. Hleihil M. Sood R. Barrera I. (2018). Local inhibition of PERK enhances memory and reverses age-related deterioration of cognitive and neuronal properties. *J. Neurosci.* 38 648–658. 10.1523/JNEUROSCI.0628-17.2017 29196323 PMC6596193

[B56] Sherdell L. Waugh C. E. Gotlib I. H. (2012). Anticipatory pleasure predicts motivation for reward in major depression. *J. Abnorm. Psychol.* 121 51–60. 10.1037/a0024945 21842963 PMC3335300

[B57] Smith W. B. Starck S. R. Roberts R. W. Schuman E. M. (2005). Dopaminergic stimulation of local protein synthesis enhances surface expression of GluR1 and synaptic transmission in hippocampal neurons. *Neuron* 45 765–779. 10.1016/j.neuron.2005.01.015 15748851

[B58] Stern E. Chinnakkaruppan A. David O. Sonenberg N. Rosenblum K. (2013). Blocking the eIF2alpha kinase (PKR) enhances positive and negative forms of cortex-dependent taste memory. *J. Neurosci.* 33 2517–2525. 10.1523/JNEUROSCI.2322-12.2013 23392680 PMC6619168

[B59] Stramiello M. Wagner J. J. (2008). D1/5 receptor-mediated enhancement of LTP requires PKA, Src family kinases, and NR2B-containing NMDARs. *Neuropharmacology* 55 871–877. 10.1016/j.neuropharm.2008.06.053 18644393 PMC2578828

[B60] Sutton M. A. Taylor A. M. Ito H. T. Pham A. Schuman E. M. (2007). Postsynaptic decoding of neural activity: eEF2 as a biochemical sensor coupling miniature synaptic transmission to local protein synthesis. *Neuron* 55 648–661. 10.1016/j.neuron.2007.07.030 17698016

[B61] Taha E. Gildish I. Gal-Ben-Ari S. Rosenblum K. (2013). The role of eEF2 pathway in learning and synaptic plasticity. *Neurobiol. Learn. Mem.* 105 100–106. 10.1016/j.nlm.2013.04.015 23742918

[B62] Tan M. L. Dyck B. A. Gabriele J. Daya R. P. Thomas N. Sookram C. (2014). Synapsin II gene expression in the dorsolateral prefrontal cortex of brain specimens from patients with schizophrenia and bipolar disorder: effect of lifetime intake of antipsychotic drugs. *Pharmacogenomics J.* 14 63–69. 10.1038/tpj.2013.6 23529008 PMC3970980

[B63] Waelti P. Dickinson A. Schultz W. (2001). Dopamine responses comply with basic assumptions of formal learning theory. *Nature* 412 43–48. 10.1038/35083500 11452299

[B64] Wang X. Li W. Williams M. Terada N. Alessi D. R. Proud C. G. (2001). Regulation of elongation factor 2 kinase by p90(RSK1) and p70 S6 kinase. *EMBO J.* 20 4370–4379. 10.1093/emboj/20.16.4370 11500364 PMC125559

[B65] Wong M. H. Samal A. B. Lee M. Vlach J. Novikov N. Niedziela-Majka A. (2019). The KN-93 Molecule Inhibits Calcium/Calmodulin-Dependent Protein Kinase II (CaMKII) Activity by Binding to Ca(2+)/CaM. *J. Mol. Biol.* 431 1440–1459. 10.1016/j.jmb.2019.02.001 30753871

