# Supplementary material for: D1 Dopamine Receptor Activation Induces Neuronal eEF2 Pathway-Dependent Protein Synthesis
Source: Front Mol Neurosci. 2020 May 15;13:67. doi: 10.3389/fnmol.2020.00067 (PMC7242790; doi:10.3389/fnmol.2020.00067)
Supplement: Supplementary file 1 [file Table_1.docx]

**
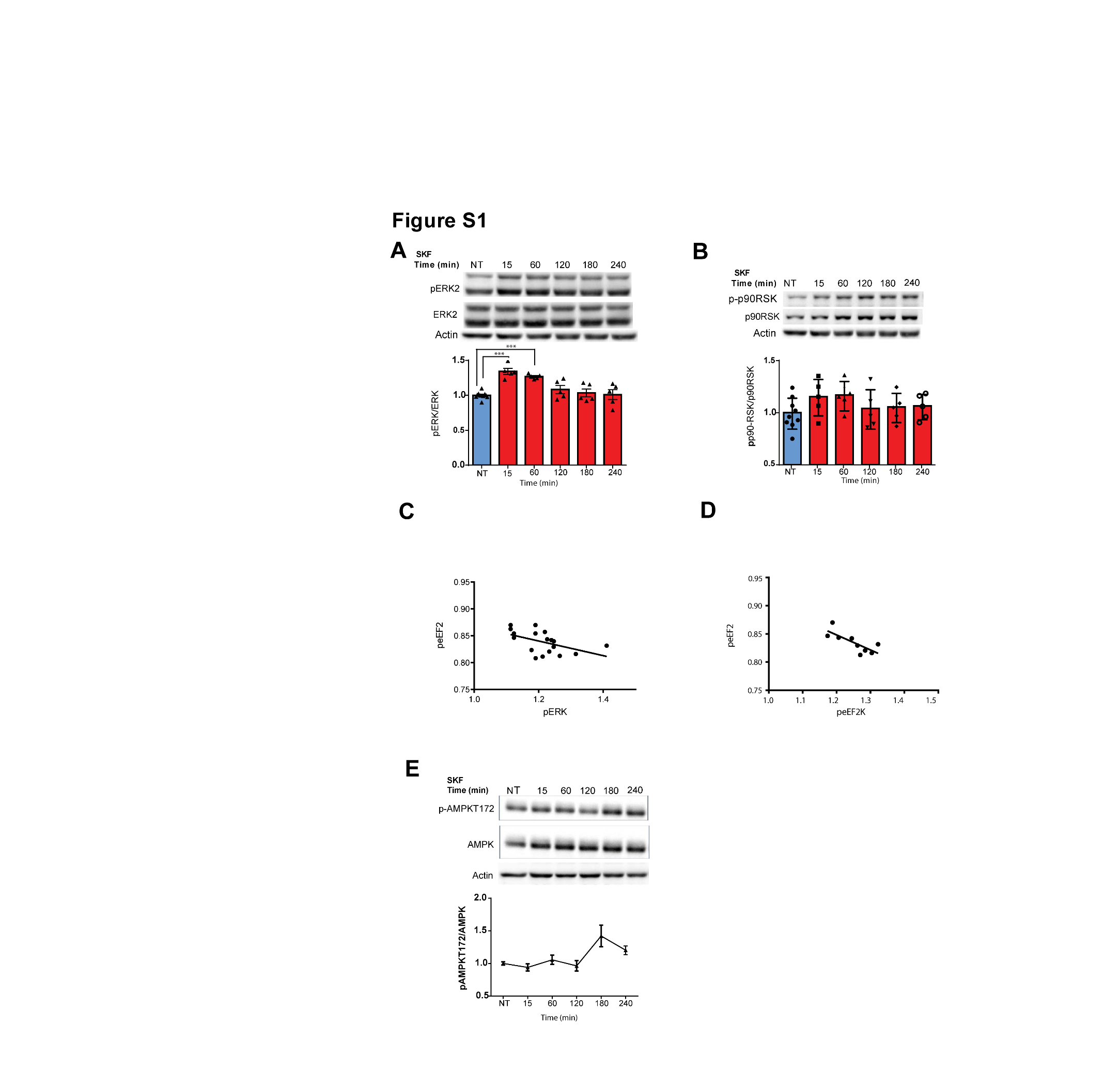
**

**Figure S1**. D1 receptor activation induces ERK1/2 phosphorylation both in vitro and in vivo and correlates with eEF2 dephosphorylation. ***(A)*** Representative blots and quantification of the ratios of phosphorylated to total ERK1/2 in cortical primary cultures incubated with D1 receptor agonist SKF38393 (25µM) for the indicated time points (from five independent cultures). One way ANOVA F_(5,29)_=11.912, p<0.0001. Post-hoc test, NT vs. SKF 15min: p<0.0001; NT vs. SKF60min: p<0.0001. ***(B)*** Representative blots and quantification of the ratios of phosphorylated to total p90RSK in cortical primary cultures incubated with D1 receptor agonist SKF38393 (25µM) for the indicated time periods (from five independent cultures). ***(C)*** Pearson's correlation of eEF2 phosphorylation levels with ERK2 phosphorylation levels. Pearson's correlation: r=-0.50, p<0.05*.****(D)*** Negative correlation between peEF2Thr^56^ and peEF2KSer^366^ in cortical cultures treated with SKF38393 for 15 min (r=-0.76, p<0.05, Pearson’s correlation). ***(E)*** Representative blots and quantification of the ratios of phosphorylated to total AMPK in cortical primary cultures incubated with D1 receptor agonist SKF38393 (25µM) for the indicated time points. One way ANOVA F(5,53)=3.008, p=0.01. Means ± SEM are shown in all graphs. *p<0.05, **p<0.001,***p<0.0001.

**
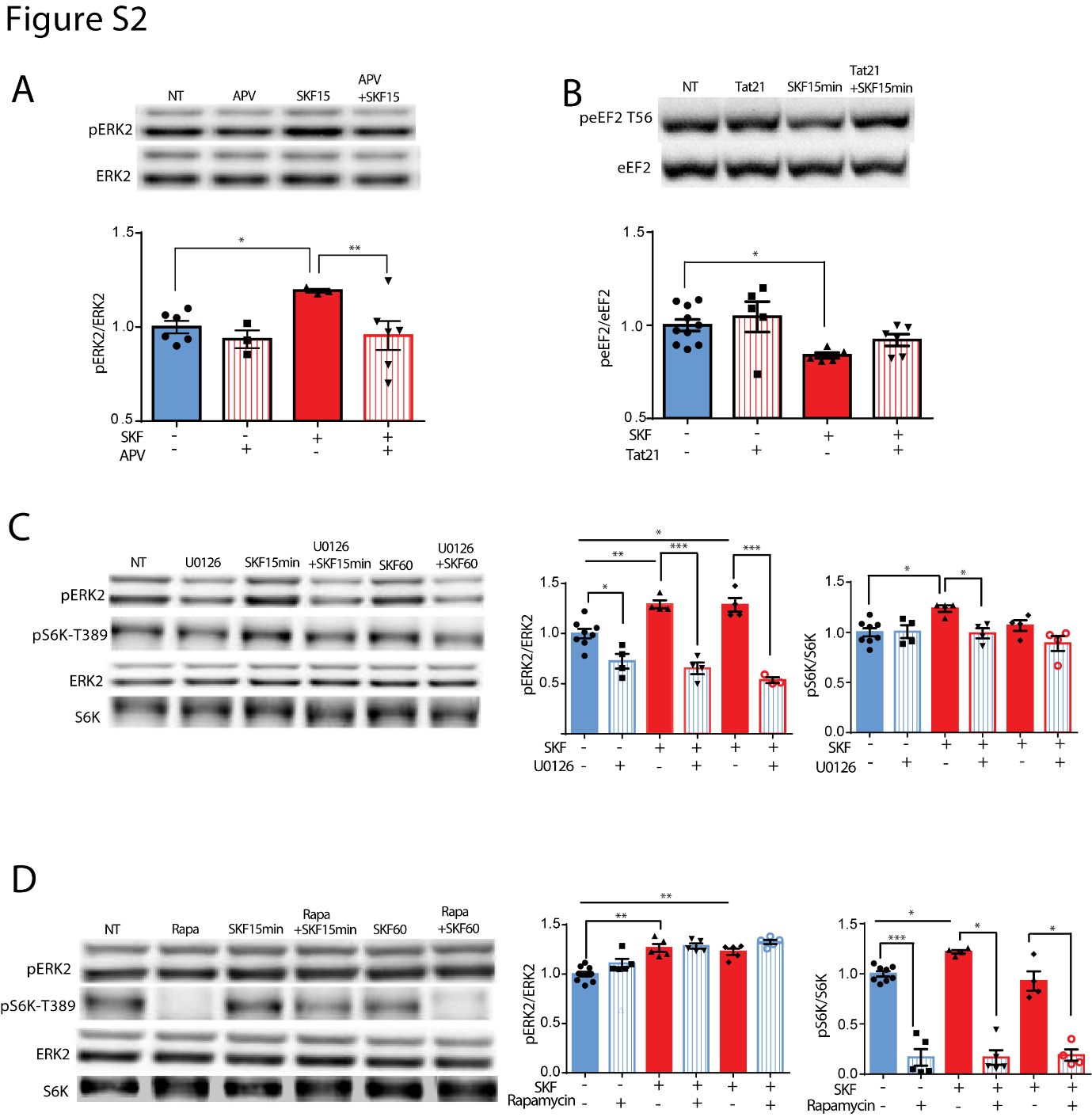
**

**Figure S2. MEK-ERK is upstream to mTOR (A)** Representative blots and quantification of the ratios of phosphorylated ERK2 (Thr202/Tyr204) to total ERK1/2 in cortical primary cultures incubated with SKF38393 (25µM) for 15 min with or without pretreatment of the NMDA receptor antagonist APV (40µM). Data are means ± SEM of five independent cultures. One way ANOVA F(3,26)= 5.830, p=0.003. Post-hoc test, NT vs. SKF 15min: p=0.03; SKF 15min vs. SKF 15min+APV: p=0.003. ***(B)*** Representative blots and quantification of the ratios of phosphorylated ERK2 (Thr202/Tyr204) to total ERK1/2 in cortical primary cultures incubated with SKF38393 (25µM) for 15 min with or without pretreatment of the CaMKII specific inhibitor TatCN21 peptide [F(3,22)= 3.89, p=0.02; Post-hoc test, NT vs. Tat21: p=0.86; NT vs. SKF 15min: p=0.04; SKF15min vs. SKF 15min+Tat21: p=0.59].

***(C)*** Representative blots and quantification of phosphorylated to total ERK1/2 and phosphorylated S6K from cortical primary cultures treated with U0126. For ERK: one-way ANOVA [F(5,21)=27.66, p<0.0001; post hoc test, NT vs. U0126: p=0.01; NT vs. SKF15: p=0.008; NT vs. SKF60: p=0.01; SKF15 vs. SKF15+U0126: p<0.0001; SKF60 vs. SKF60+U0126: p<0.0001] for S6K One-way ANOVA [F(5.22)=4.179, p=0.008; post hoc SKF15 vs NT: p=0.02; SKF15 vs SKF15+U0126: p=0.04]. ***(D)*** Representative blots and quantification of phosphorylated to total ERK2 and phosphorylated S6K from cortical primary culture treated with rapamycin. For ERK: One-way ANOVA [F(5,29)=19.99, p<0.001; post hoc test, NT vs. SKF15: p<0.001;NT vs. SKF60: p<0.001; NT vs. SKF60+rapa: p<0.001]. For S6K One-way ANOVA [F(5,24)=63.86, p<0.001; post hoc test, NT vs. rapa: p<0.0001; NT vs. SKF15: p=0.03; SKF15 vs. SKF15+rapa: p<0.0001; SKF60 vs. SKF60+rapa: p<0.0001]. Means ± SEM are shown in all graphs. *p<0.05, **p<0.001,***p<0.0001.

**
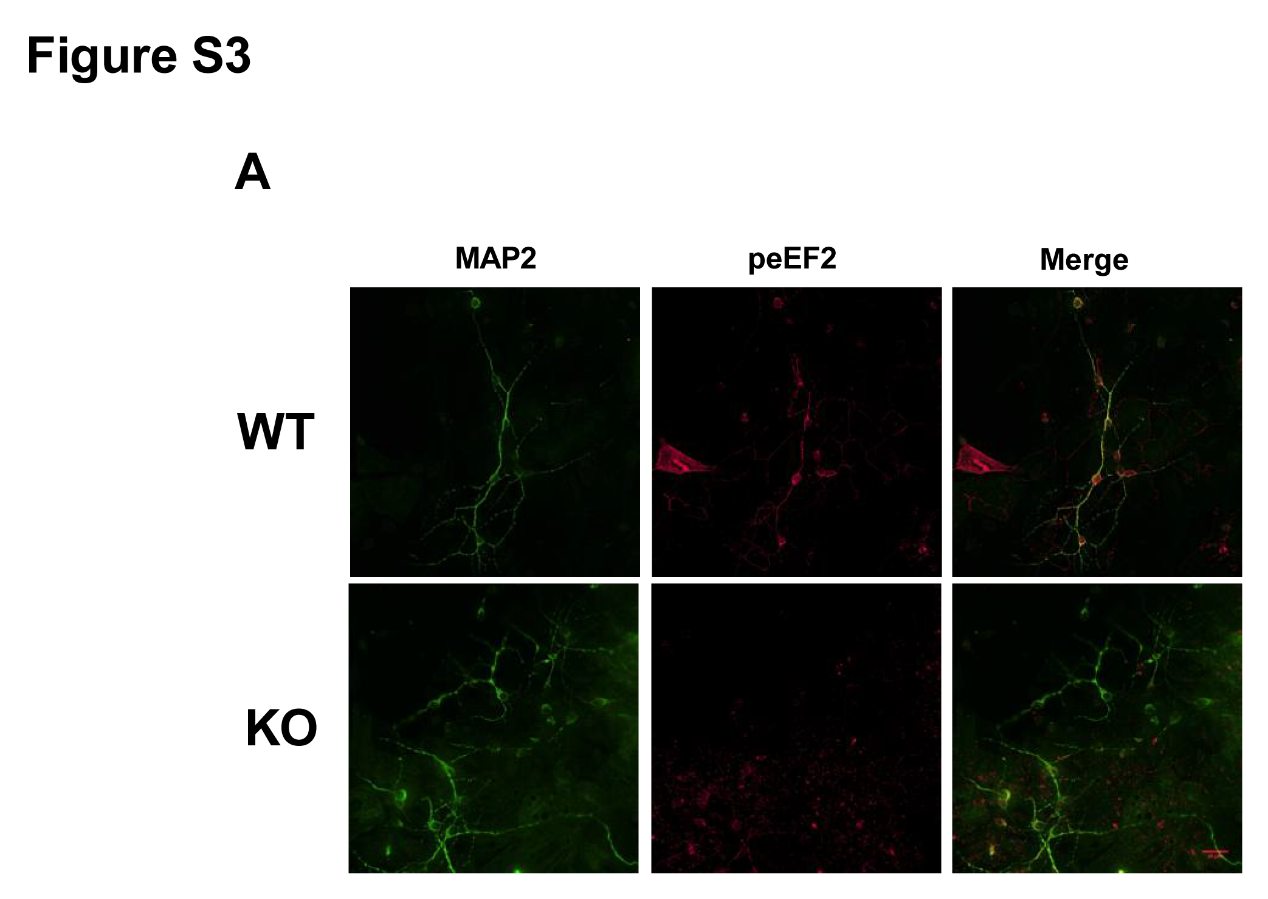
**

**Figure S3.** Related to main figure 3. Representative images of phospho-eEF2 in primary cultures from WT and eEF2-KO mice. Immunofluorescence using phospho-eEF2 (red) and MAP2 (green) antibodies in cortical primary cultures from WT and eEF2K-KO mice. Scale bar 20µm.


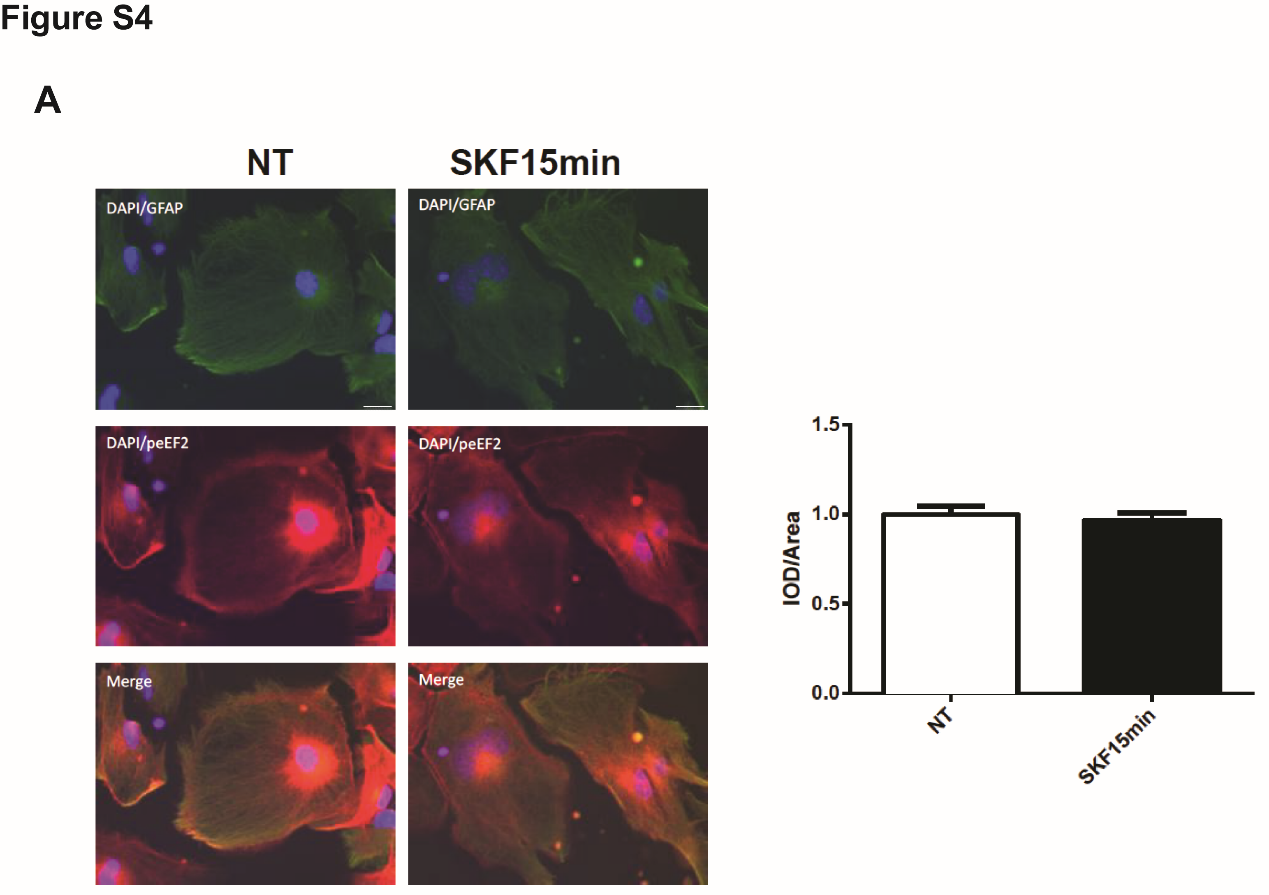


**Figure S4.** Dopamine D1 receptor activation has no effect on eEF2 phosphorylation in glia cells. Representative images of phospho-eEF2 in glia cells from primary cultures. Immunofluorescence using phospho-eEF2 (red) and GFAP (green) antibodies in cortical primary cultures from C57/Bl6 mice. Scale bar 20µm.


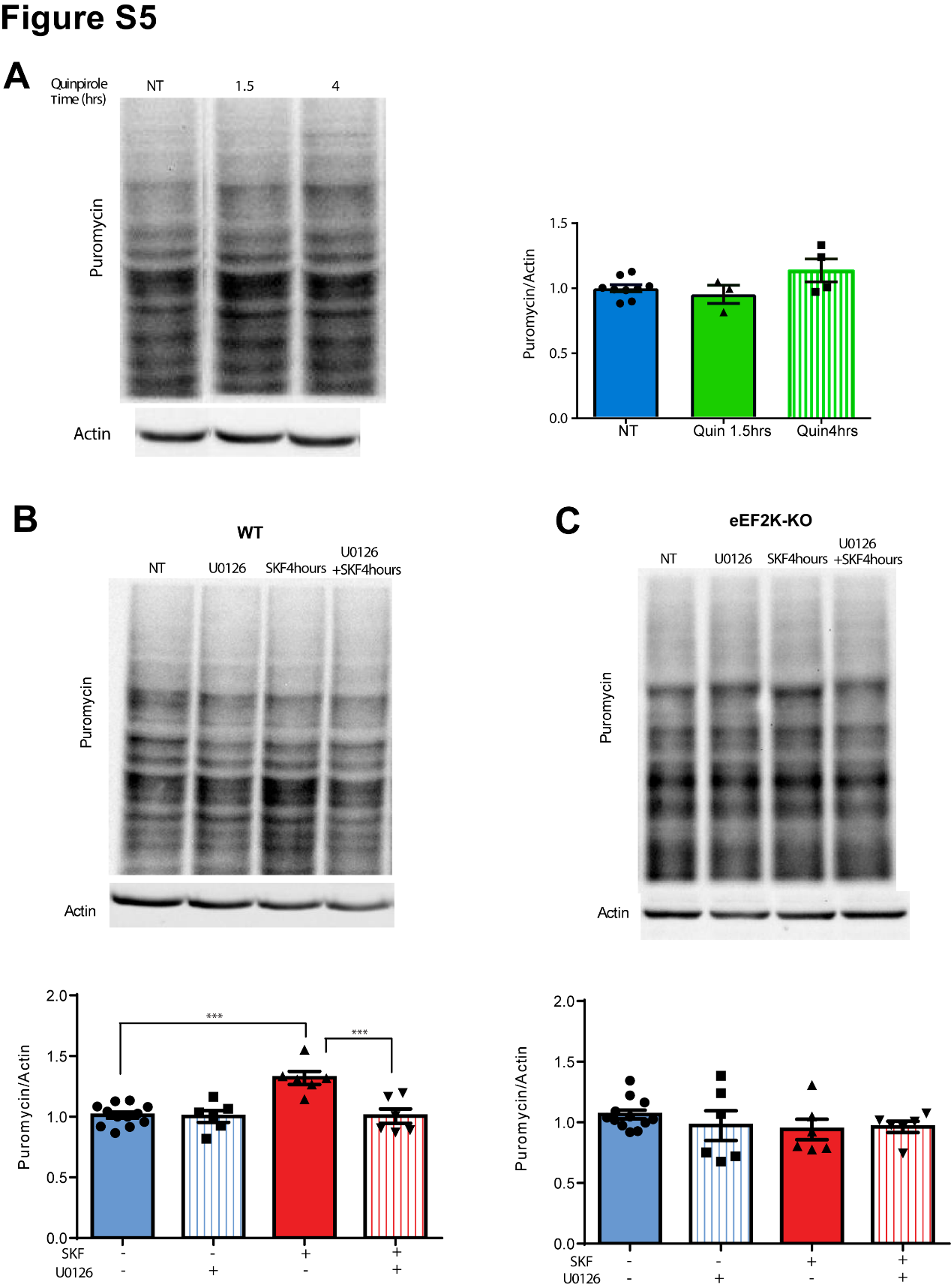


**Figure S5.** MEK/ERK inhibition reduces D1 receptor-dependent de novo protein synthesis increases in WT but not eEF2K-KO mice. ***(A)*** Representative Western blots and quantification of proteins labeled with puromycin in C57/Bl6 primary cortical neurons treated with quinpirole (10µM) for 1.5 or 4 hours. Puromycin signal was quantified and normalized to β-actin loading control. One-way ANOVA F(2,11)=1.292, p=0.313. ***(B-C)*** WT or eEF2K-KO mouse-derived cortical neurons were pre-treated with MEK inhibitor U0126 (20µM) (or vehicle) for 30 min followed by 4 hours of incubation with SKF38393 (25µM). Cells were treated for 10 min with puromycin (1µg/ml) before harvesting. Puromycin incorporation was detected by Western blotting. Data are means ± SEM of six independent cultures. *p<0.05. For WT: One way ANOVA F_(3,26)_=11.76, p<0.001. Post-hoc test, NT vs. SKF4h: p<0.001; SKF4h vs. SKF4h+U0126: p<0.001. For KO: F_(3,26)_=0.79, p=0.51.
