# Supplementary material for: D1 Dopamine Receptor Activation Induces Neuronal eEF2 Pathway-Dependent Protein Synthesis
Source: Front Mol Neurosci. 2020 May 15;13:67. doi: 10.3389/fnmol.2020.00067 (PMC7242790; doi:10.3389/fnmol.2020.00067)
Supplement: Supplementary file 2 [file Table_2.DOCX]

Figure 1-Source data- Relevant to Fig1.





Figure 2-Source data1- relevant to Fig. 2 A and B.





Figure 2-Source data2- Relevant to Fig. 2 C





Figure 2-Source data3- Relevant to Fig. 2 D


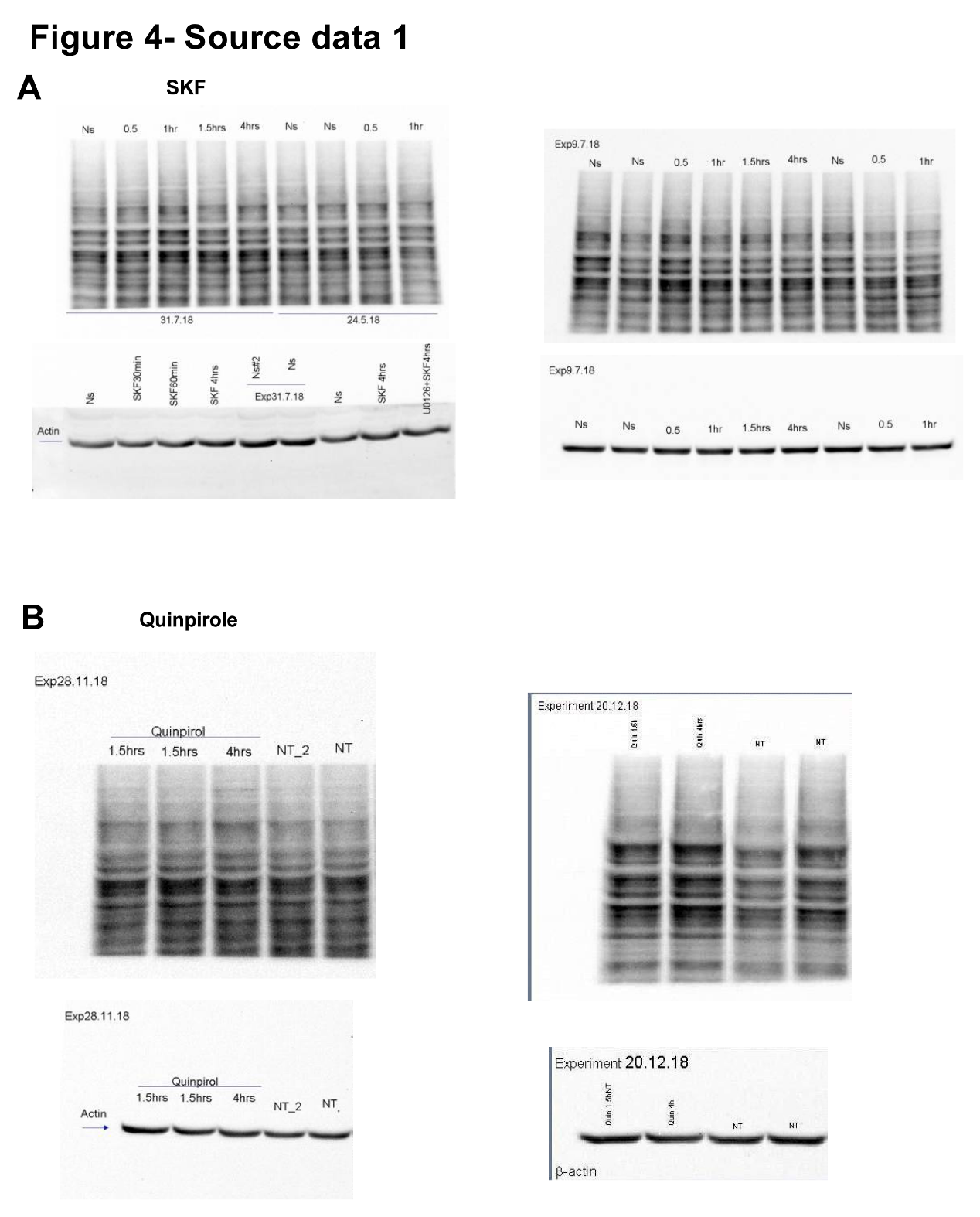


Figure 4-Source data 1- Relevant to Fig. 4 A and B


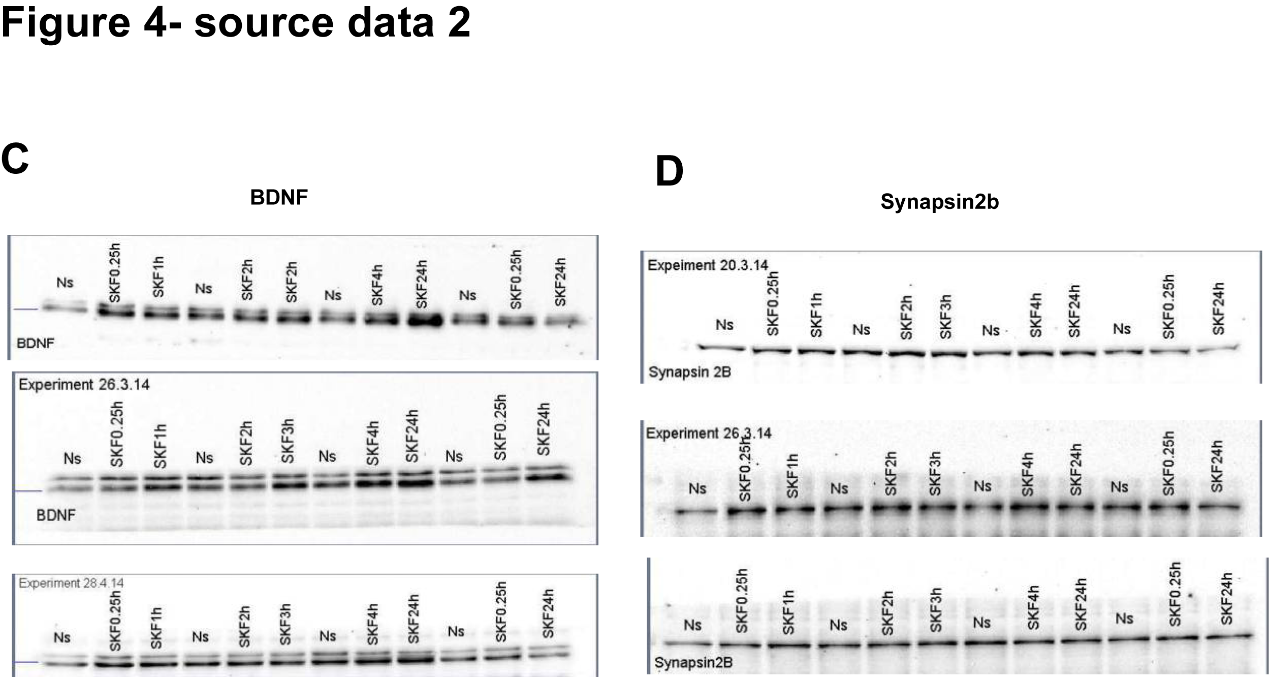


Figure 4-Source data 2- Relevant to Fig. 4 C and D





Figure 5-Source data- Relevant to Fig. 5 A and B


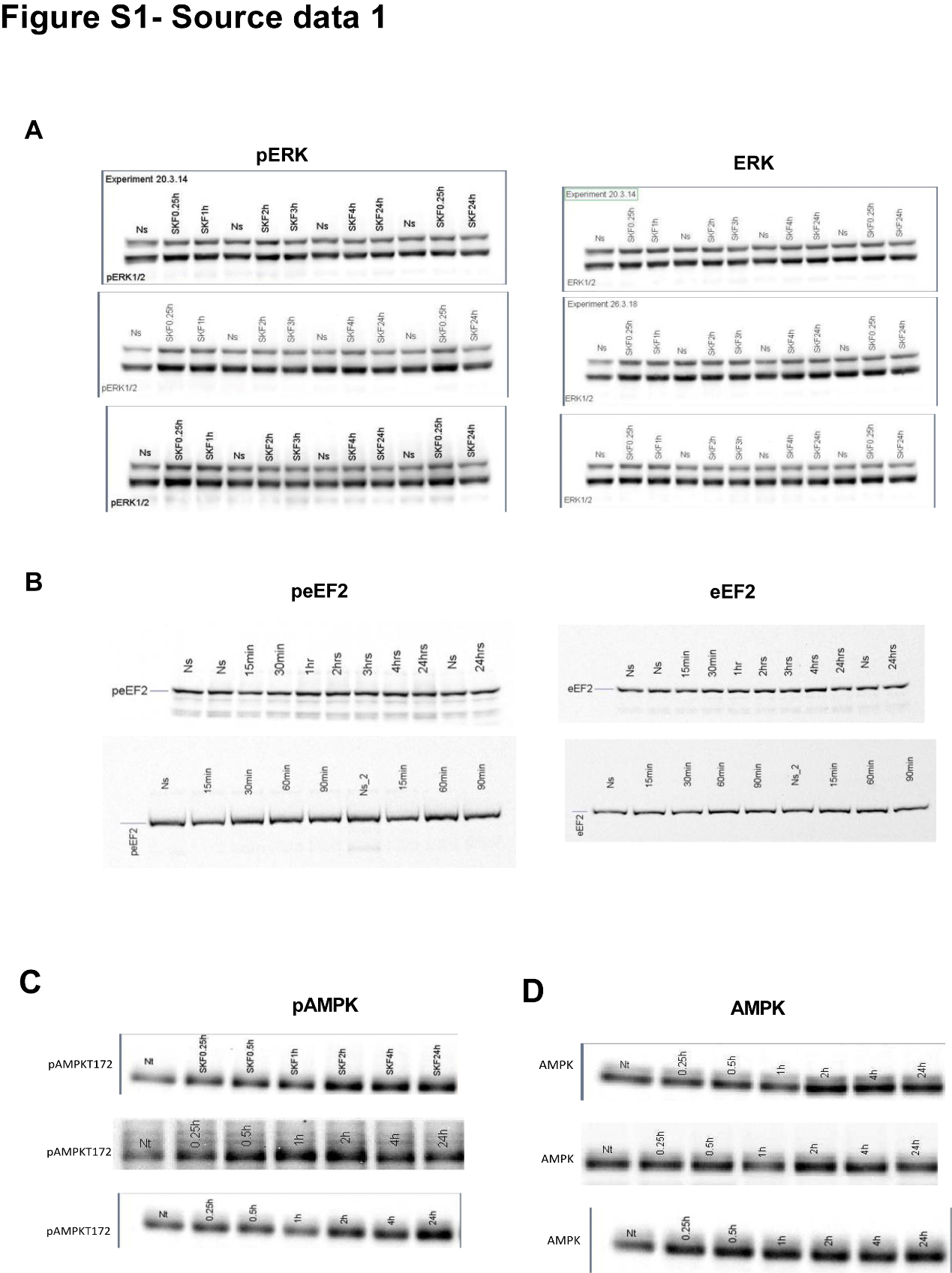


Figure S1-Source data- Relevant to fig. S1 A-D.





Figure S2-Source data1- Relevant for Fig. S2 A and B





Figure S2-Source data2- Relevant for Fig. S2 C





Figure S2-Source data3- Relevant for Fig. S2 D





Figure S5-Source data- Relevant for Fig. S5 A,B and C.
